# Learning and Choice in Mood Disorders: Searching for the Computational Parameters of Anhedonia

**DOI:** 10.1162/CPSY_a_00009

**Published:** 2017-12-01

**Authors:** Oliver J. Robinson, Henry W. Chase

**Affiliations:** 1 Institute of Cognitive Neuroscience, University College London, London, UK; 2 Department of Psychiatry, University of Pittsburgh School of Medicine, Pittsburgh, PA, USA

**Keywords:** reinforcement learning, mood disorders, anxiety, decision making, computational psychiatry

## Abstract

Computational approaches are increasingly being used to model behavioral and neural processes in mood and anxiety disorders. Here we explore the extent to which the parameters of popular learning and decision-making models are implicated in anhedonic symptoms of major depression. We first highlight the parameters of reinforcement learning that have been implicated in anhedonia, focusing, in particular, on the role that choice variability (i.e., “temperature”) may play in explaining heterogeneity across previous findings. We then turn to neuroimaging findings implicating attenuated ventral striatum response in anhedonic responses and discuss possible causes of the heterogeneity in the literature. Taken together, the reviewed findings highlight the potential of the computational approach in teasing apart the observed heterogeneity in both behavioral and functional imaging results. Nevertheless, considerable challenges remain, and we conclude with five unresolved questions that seek to address issues highlighted by the reviewed data.

## INTRODUCTION

Mood and anxiety disorders are a major worldwide health burden across individual, social, and economic levels (Beddington et al., 2008). Despite a number of effective therapies and growing neuroscientific understanding of disease processes, resistance to established treatment strategies remains high (Yonkers, Warshaw, Massion, & Keller, 1996). This may be, at least in part, because current clinical diagnoses of these disorders rely primarily on *subjective* symptoms and behaviors, while the goal of neuroscience is to understand *objective* (i.e., observer-independent) biological mechanisms. Mapping these two approaches onto one another is exceptionally difficult and fraught with potential bias but is ultimately critical if we want to improve our ability to develop new treatments and target current treatments more effectively. To this end, it has been suggested that computational modeling of behavior—the focus of this review—can provide a means of bridging the gap between observable symptoms and behavior to underlying neurobiological mechanisms (Huys, Maia, & Frank, 2016; Montague, Dolan, Friston, & Dayan, 2012).

### Computational Modeling of Behavior

Computational models of behavior offer a large and powerful explanatory repertoire with the potential of integrating information derived from a variety of different sources (e.g., electrophysiology, neuroimaging, behavior) into a coherent theoretical structure. Applying such models to psychiatric disorder symptomology offers a number of advantages:

1.  Models require*hypotheses to be explicitly quantified*. That is to say, experimental design and analysis require an explicit proposal of what is driving behavior or neural activity. Rather than specifying that major depression is associated with, say, “negative affective bias” (Roiser, Elliott, & Sahakian, 2012), the component parts driving that bias must be specified. For instance, one must specify if a single parameter drives sensitivity to reward and punishment processing (i.e., whether an individual dislikes punishments to the exact same degree that he or she likes rewards) or whether these should be considered separate processes (allowing particularly extreme dislike of punishments with ambivalence toward rewards, for example).
2.  Although “all models are wrong” (Box, 1976), some are more wrong than others, and evaluation of the components that improve model fit in a model comparison procedure can *formally assess the relative strengths of competing hypotheses to explain a given dataset.* For example, the relative explanatory validity of single versus separate reward and punishment sensitivity parameters can be directly compared. Simulations and comparison across datasets can then provide further support for or against a given model (Palminteri, Wyart, & Koechlin, 2017).
3.  Rather than relying on summary mean or variance statistics, models can be used to explore trial-by-trial variance. This is particularly important when studying cognitive and learning processes (which are strongly implicated in many psychiatric disorders, including major depression; Rock, Roiser, Riedel, & Blackwell, 2014) in which subjects’ behav ior can change dynamically through the task. This *temporally rich approach explores variance over time and thus obtains more information from a given dataset.* Indeed, subtle effects that are often overwhelmed in collapsed mean accuracy or response times can only be revealed using the modeling approach (White, Ratcliff, Vasey, & McKoon, 2010).
4.  Models can be constrained by our understanding of what is biophysically plausible given our understanding of neuronal and pharmacological interactions. Combined with neuroimaging, winning model parameters can provide us with a means of *mechanistically linking observable behavior and underlying neural substrates.*
5.  *Models can show emergent properties,* and explanations that might otherwise be considered excessively complex can be specified and tested. A good example of this is where *meta parameters*—or variance of model parameters—are implicated in pathologies. For instance, trait anxiety is associated with reduced learning rate *adaptability* (Browning, Behrens, Jocham, O’Reilly, & Bishop, 2015). Specifying and identifying this alteration without a model would be more difficult from both practical and conceptual standpoints.

**Figure 1.**
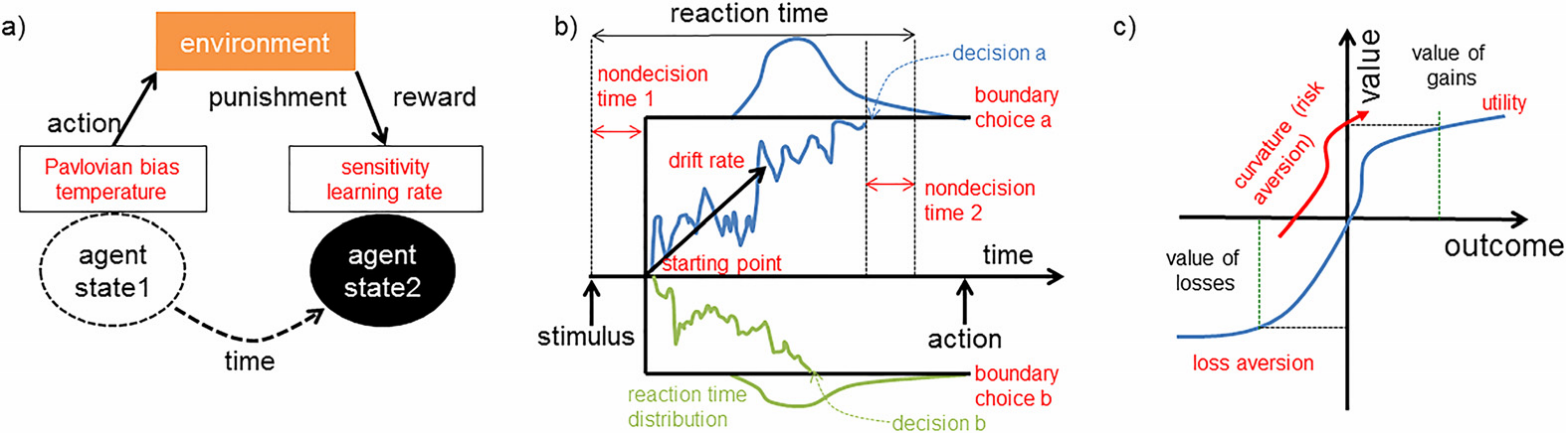
**Schematic of the learning and choice models discussed in the article.** a) Illustrative example of reinforcement learning models. b) Illustrative example of reaction time models (e.g., drift diffusion models). c) Illustrative example of models of economic choice under uncertainty.

In this review, we focus on the application of computational modeling to anhedonia—diminished reward processing—in major depression. We review findings from two primary classes of models (Figure 1) that have been implicated in anhedonia (and, to a lesser extent, in anxiety, which is often comorbid with depression): (a) reinforcement learning models and (b) reaction time models. We also briefly refer to (c) models of economic choice under uncertainty, as they allow us to illustrate a point about the importance of temperature (see section “Simulation Showing the Importance of Temperature in Decision-Making Models”). Notably, although we consider them separately here, these models can also be combined to generate more complicated models (Pedersen, Frank, & Biele, 2016).

#### Reinforcement learning models

*Reinforcement learning* (RL; Figure 1A) models seek to explain choice behavior based on response to rewards and punishments received from the environment. Specifically, organisms are thought to respond to deviations in expectancy about rewards and punishments based on a prior belief about how likely an event is to occur. Each occurrence of a stimulus–outcome pairing increases the expectancy of that outcome and gradually reduces *prediction error* (PE). The PE for the current trial is the actual outcome value of the current trial compared to the *expected* value of the outcome (Bush & Mosteller, 1955; Rescorla & Wagner, 1972). The expected value is calculated by adding the value of the previous trial to the PE of the previous trial multiplied by the rate at which an individual learns the new associ ation, known as the learning rate (Equation 2); Sutton & Barto, 1998). This value calculation can also be influenced by Pavlovian biases that encourage a bias toward making actions that lead to reward and inhibiting actions that lead to punishment (as an evolutionarily efficient means of maximizing rewards and minimizing punishments (Equation 3); Guitart-Masip et al., 2011; Huys, Golzer, et al., 2016). How these parameters may be related to symptoms of mood disorders are reviewed in Table 1. To compile this table, we primarily focused on salient exemplars of particular paradigms and analysis techniques. However, we also performed a literature search to include as many examples of data consistent or inconsistent with the construct in question as possible.

**Table 1.** Reinforcement learning model parameters that could be altered in anhedonia

**Construct**	**Description**	**Computational instantiation**	**Evidence implicating**	**Evidence exonerating**	**Missing evidence**
Value-guided behavior	Capacity of value representations to guide choice	Value (Equation 1)		Most studies report broadly intact acquisition	
Feedback insensitivity	“Blunted” response to feedback, both positive and negative	Reduced learning rate (Equation 2)	Chase, Frank et al. (2010), Steele et al. (2007)	Rothkirch, Tonn, Kohler, & Sterzer (2017)	
Enhanced punishment sensitivity	Relatively enhanced response to negative feedback	Enhanced learning rate if outcome is aversive	Beevers et al. (2013), Herzallah et al. (2013), Maddox et al. (2012), Murphy, Michael, Robbins, & Sahakian (2003), Taylor Tavares et al. (2008)	Cavanagh, Bismark, Frank, & Allen (2011), Chase, Frank et al. (2010), Whitmer, Frank, & Gotlib (2012)	
Reduced reward sensitivity	Relatively reduced response to positive feedback	Reduced learning rate if outcome is appetitive	Beevers et al. (2013), DelDonno et al. (2015), Herzallah et al. (2013), Kunisato et al. (2012), Maddox et al. (2012), O. J. Robinson et al. (2012), Treadway, Bossaller, Shelton, & Zald (2012)	Cavanagh et al. (2011), Chase, Frank et al. (2010), Chase, Michael, Bullmore, Sahakian, & Robbins (2010), Whitmer et al. (2012)	
Pavlovian bias	Influence of reward- or punishment-predictive stimuli on behavior	See Equation 3	Bylsma, Morris, & Rottenberg (2008), Huys, Golzer et al. (2016), Radke, Guths, Andre, Muller, & de Bruijn (2014); see Mkrtchian, Aylward, Dayan, Roiser, & Robinson (2017) for anxiety		
Temperature	Stochastic choice	Temperature (Equation 4)	Huys et al. (2012), Huys et al. (2013), Kunisato et al. (2012); for indirect evidence, see Blanco, Otto, Maddox, Beevers, & Love (2013), Clery-Melin et al. (2011); for trend level, see Chase et al. (2017)	Chung et al. (2017), Rothkirch et al. (2017)	
Reduced outcome magnitude sensitivity	Linear or nonlinear scaling of utility across increasing expected value	[Outcome*sensitivity] or [Outcome^sensitivity]	Indirect evidence: Herzallah et al. (2013), Treadway et al. (2012)		
Effort costs	Suppression of responding by effort	[Outcome value–effort cost]	Hershenberg et al. (2016), Treadway et al. (2012), Yang et al. (2016), Yang et al. (2014)	No simple increase in effort costs: Clery-Melin et al. (2011), Sherdell, Waugh, & Gotlib (2012)	
Working memory/“model-based” learning	Rapid adaptation of behavior in response to feedback	Various approaches, e.g., control choice in terms of previous outcome (Myers et al., 2016)	N/A	N/A	Little direct examination in MDD
Uncertainty-modulated learning	Increases or decreases in learning rate in response to uncertainty	Modulation of learning rate (e.g., Equation 2) by stimulus/outcome uncertainty	N/A	N/A	Little direct examination in MDD (but see Browning et al., 2015, on anxiety)

*Note.* Here we define indirect evidence as suggestive that the construct might be significant, but this was not assessed directly via a modeling or other analytic strategy. To complete this table, combinations of the following terms were used in systematic searches: *reward, model-based learning, Pavlovian, exploration, decision, choice, punishment learning,* with *anhedonia* or *major depression.* The goal of the table is to provide an overview of salient exemplars of existing data from studies incorporating depressed, dysphoric, or euthymic individuals, which may be particularly relevant for the constructs listed.

#### Reaction time models

While RL models are concerned with decision making and choices, diffusion models try to explain the distribution of reaction times to make those choices (Tsetsos, Gao, McClelland, & Usher, 2012; Figure 1B). There are a number of different frameworks, but most share the fundamental concept that information is accumulated (they are sometimes referred to as *accumulator models*) until a threshold is reached and a decision is made (Ratcliff, Smith, Brown, & McKoon, 2016; Tsetsos et al., 2012). The parameters in such models generally include (a) how far apart the decision options are (boundaries), (b) the rate at which a decision speeds toward the boundaries (drift rate), (c) whether the individual has a bias toward one or another of the options (starting point between the boundaries), and (d) the time to encode the stimuli and process a motor response (nondecision time; White et al., 2010). These parameters are then used to explain the distributions of reaction times for one decision over another (they are most commonly used for tasks in which participants have to decide between one of two responses).

#### Models of economic choice under uncertainty

Economic models of decision making can describe adaptive choice when options are uncertain or subject to various costs (Figure 1C). In general, most individuals overweigh losses relative to equivalent gains and show a preference for certain over-risky outcomes with equal or higher expected value. Within this framework, economic decisions can be explained by a combination of two factors: reduced sensitivity to outcome value as value increases (i.e., risk aversion) and an overweighting of losses relative to gains (i.e., loss aversion; Charpentier, Aylward, Roiser, & Robinson, 2016; Sokol-Hessner et al., 2009; Tversky & Kahneman, 1992).

There is limited work exploring these models in depression (but see Beevers et al., 2013; Maddox, Gorlick, Worthy, & Beevers, 2012), so we do not review their relationship with anhedonia here. Rather, we use this model to make a point about the temperature parameter that is more clearly illuminated in the absence of the learning that is inherent in RL models (see section “Simulation Showing the Importance of Temperature in Decision-Making Models”). In other words, although we focus primarily on RL as a paradigmatic example of a broader decision-theoretical approach to psychopathology (Montague, 2012), the theoretical and practical issues raised may be broadly applicable across paradigms (e.g., loss or risk aversion but also temporal discounting, Lempert & Pizzagalli, 2010; Pulcu, Trotter et al., 2014, and social decision making, Gradin et al., 2015; Pulcu, Zahn, et al., 2014).

### Anhedonia and Impairments of Reward-Directed Behavior

A recurrent feature of a variety of psychiatric disorders is a “loss of interest or pleasure” in previously enjoyable activities. This phenotype is referred to as anhedonia and is particularly prevalent in disorders such major depression, schizophrenia (SZ), and addiction (Franken, Rassin, & Muris, 2007). Here we focus on major depression, in which anhedonia is a key symptom used for diagnosis. Although anhedonia might be straightforwardly characterized in terms of a reduction in response (across cognitive domains) to rewarding events, many features of the phenotype remain puzzling (Pizzagalli, 2010). For instance, it has been argued that anhedonia should be broken down into *anticipatory*, *decisional*, and *consummatory* components (Argyropoulos & Nutt, 2013; Treadway & Zald, 2011) as well as further distinct disturbances in the *social*domain (Christianson et al., 2008). Within these categories, responses to certain kinds of reinforcers might be selectively altered (e.g., affective responses to music; Martinez-Molina, Mas-Herrero, Rodriguez-Fornells, Zatorre, & Marco-Pallares, 2016).

Anhedonia is often measured, for both experimental and clinical purposes, using self-report questionnaires and interviews (Rizvi, Pizzagalli, Sproule, & Kennedy, 2016). Although these tools are frequently psychometrically reliable, they suffer from prominent limitations, namely, demand characteristics (i.e., individuals give you the answer they think you expect) and anchoring effects (i.e., individuals give answers consistent with the first answer they gave). Indeed, as highlighted by the memory literature, an individual’s pattern of behavioral responses on cognitive tasks can be a more reliable proxy of the degree of memory encoding than self-report (Shanks & St. John, 1994; Vadillo, Konstantinidis, & Shanks, 2016). Consistent with this is evidence that behavioral measures of anhedonia can vary independently from self-reported anhedonia (Pechtel, Dutra, Goetz, & Pizzagalli, 2013). Moreover, despite clear differences in self-reported anhedonia, prior reviews have yielded little evidence of substantial differences between depressed and healthy individuals in terms of their hedonic responses on consummatory tests (Treadway & Zald, 2011). For instance, the pleasure-evoking properties of sweet tastes are generally similarly rated by depressed and healthy individuals (Treadway & Zald, 2011). This discrepancy is somewhat perplexing—if anhedonic individuals describe a selective lack of interest in rewarding experiences, why is this not borne out in their behavioral responses? The computational approach might help resolve this question. For instance, it may be that, while the end points (e.g., response to sweet tastes) appear identical in anhe donic individuals, the individual components of the mechanisms (e.g., detection of, or learning about, the tastes) that lead to this end point differ. The anhedonic individual may take longer, or require more evidence, to reach the same end point. These effects will be hidden if one measures the end point alone: Computational models enable the specification and exploration of these hidden component parts.

## APPLYING COMPUTATIONAL APPROACHES TO BEHAVIORAL FINDINGS IN ANHEDONIA

### Sensitivity to Value

One way to consider a deficit in reward-oriented behavior is to argue that individuals are able to learn associations between actions and reinforcers but fail to use this information to guide behavior. A typical choice rule (Equation 1), also referred to as an *observation model*, describes the probability of eliciting a response (i.e., action probability) given an action’s value using what is known as a *softmax function*:

$${Action\;Probability}_{\left\{s_{t}\}(a\right)}=\left[\frac{\text{exp}\left({Value}_{\left\{s_{t}\}(a\right)}\right)}{\sum_{b=1}^{n}\text{exp}\left({Value}_{\left\{s_{t}\}(b\right)}\right)}\right].$$ (1)

The value that is entered into this equation needs to be acquired from the environment by the individual. This is commonly described via a RL process where outcomes are positive for gains and negative for losses and, crucially, that includes a free parameter, called *sensitivity*, that describes how much weight an individual ascribes to those outcomes:

$${Value}_{\left\{s_{t}\}(a_{t}\right)}⇐{Value}_{\left\{s_{t}\}(a_{t}\right)}+LearningRate\cdot \left(\left(Sensitivity\cdot {outcome}_{\left\{t\right\}}\right)-{Value}_{\left\{s_{t}\}(a_{t}\right)}\right).$$ (2)

Looking at Equation 1, it is possible to see that if the value is low, then the probability of a response will be diminished. So if anhedonia reduces value—perhaps by decreasing the influence of outcome through the sensitivity parameter—a natural prediction is that it would suppress responding.

In two alternative forced-choice (2AFC) cognitive tasks frequently used to study RL, a participant will have no option but to accept one option, so two equally (un)attractive options will lead to essentially random responding, because neither is favored. In go/no-go tasks—where the alternative choice is to do nothing (no-go)—this would lead to random decisions to wait (or, in the case of animal models, engage in grooming behavior). Broadly, however, the empirical evidence has not strongly supported a clear role of reduced sensitivity to value in anhedonia. There are numerous examples of intact or at least adequate instrumental control by reinforcers: The majority of studies in Table 1 report broadly compatible task acquisition across healthy and depressed cohorts.

### Learning Rate

While patients with depression can show moderate deficits in memory (Rock et al., 2014), they typically show broadly intact acquisition of the basic reward contingencies employed in RL tasks, indicating intact learning processes. Computational methods provide some support for this view, with tasks that are sensitive to anhedonia showing intact task acquisition learning (Huys, Pizzagalli, Bogdan, & Dayan, 2013). Moreover, tasks which have designed to isolate specific learning rates for rewards and punishments (i.e., where the learning rate is different for rewarding and aversive outcomes, respectively; see Equation 2) have provided mixed data but have frequently failed to support diminished reward learning or heightened punishment learning (see Table 1). For example, in the case of the Probabilistic Selection Task (PST), one study showed similar rates of positive and negative learning between controls and patients (Chase, Frank et al., 2010) and a second showed relatively enhanced positive learning in low- but not high-dysphoric individuals (Kunisato et al., 2012), while two further studies showed complex findings depending on trial type (Cavanagh et al., 2011) or rumination induction (Whitmer et al., 2012). Finally, a theoretically compatible but somewhat distinct procedure revealed asymmetrical learning rates as well as an influence of medication (Herzallah et al., 2013). Thus, when taken together, the complexity of these findings argues against a simple alteration of learning rates.

In some cases, differences in performance may emerge because individuals adopt a different learning model from that outlined in Equation 2. Individuals may vary in their tendency to change behavior on the trial immediately after unexpected feedback. Within the basic RL framework, this might be driven by a heightened (Murphy et al., 2003) or by a diminished (Chase, Frank et al., 2010; Steele, Kumar, & Ebmeier, 2007) impact of the previous trial. The broader interpretation of this type of feedback sensitivity is a topic of active debate: Recent computational approaches sometimes posit a *win–stay, lose–shift* parameter (den Ouden et al., 2013; Myers et al., 2016), which is, in essence, a simplistic *1-back* learning model and may reflect the function of working memory (Collins & Frank, 2012). This representational system does not keep track of reinforcement history beyond a very restricted number of recent outcomes (e.g., one). This approach can work well in some environments but will perform poorly on tasks (such as the commonly used Iowa Gambling Task; IGT) in which the most rewarding choice is also associated with large, if occasional, losses and in which incremental learning is necessary.

It is possible that these sorts of decision-making strategies (as well as different exploratory strategies; Knox, Otto, Stone, & Love, 2011), rather than a more fundamentally impaired learning rate mechanism, are responsible for the relatively subtle deficits that are observed in patient groups. Critically, the adoption of such strategies does not preclude intact learning: They may simply reflect selection of a strategy that will perform favorably in deterministic designs but suboptimally in others (Collins, Brown, Gold, Waltz, & Frank, 2014). Further support for this position is the presence of deficits on tasks with shifting contingencies, such as probabilistic reversal learning (Murphy et al., 2003) and the IGT (Must et al., 2006), performance on which may be particularly dependent on such strategies (e.g., den Ouden et al., 2013). Some modeling approaches (Collins & Frank, 2012; Myers et al., 2016) have therefore sought to combine these two approaches, with both RL and *win–stay*, *lose–shift* components in the same model, combined with a free parameter to determine the likelihood of one or another when the two systems come into conflict (e.g., straight after negative feedback). Overall, however, clear evidence for altered learning rates in anhedonia across a variety of paradigms is lacking.

### Pavlovian Bias

Learned or prepotent Pavlovian biases can represent powerful decision-making shortcuts that can influence RL. Specifically, *approaching* rewards and *avoiding* punishments, or stimuli which may predict them, consitutes a simple behavioral heuristic that will serve well in a wide range of scenarios. These adaptive behavioral heuristics are often referred to as *Pavlovian biases*. Pavlovian instrumental transfer (PIT) describes the influence that these biases can have over instrumental control (e.g., the simple goal-directed behaviors described by Equations 1 and 2). Experimentally, this manifests as, for example, making it extremely difficult for both humans and animal models to “approach” (e.g., make a go response to) a cue that has previously been associated with punishment (Guitart-Masip et al., 2011). Cavanagh, Eisenberg, Guitart-Masip, Huys, and Frank (2013) have operationalized Pavlovian influence over RL as a multiplier on stimulus value (and is often represented by an independent, additive parameter to that representing action or instrumental value: cf. Holland, 2004):

$${Value}_{\left\{s_{t}\}(go\right)}^{\prime }=PavlovianBias\cdot {Value}_{\left(s_{t}\right)}+Value_{\left\{s_{t}\}(go\right)}.$$ (3)

Where ${Value}_{\left(s_{t}\right)}$ is a state value, while ${Value}_{\left\{s_{t}\}\{a\right\}}$ is a state-action value-both being independently learned by a prediction error mechanism (i.e., following the form of Equation 2). *Value*′ determines choice via a softmax equation (e.g., Equation 1).

If the value (see Equation 2) is positive (i.e., rewarding), then this bias parameter increases value, whereas if the value is negative (punishing), this parameter serves to decrease value. Examining PIT, Huys, Golzer, et al. (2016) observed a reduced influence of Pavlovian bias on approach and avoidance behavior in depressed individuals compared to healthy controls (HC). Similarly, work has demonstrated that an approach bias for positive stimuli and an avoidance bias for negative stimuli in HC—that is, Pavlovian bias—is absent in depressed individuals (Radke et al., 2014). In other words, although overall instrumental behavior is generally intact in depression, the influence of *incidental*Pavlovian cues on behavior may be reduced. These findings from PIT or PIT-like paradigms may also be relevant for interpreting data from emotional cues more generally: A meta-analysis of responses to emotional cues, both positive and negative, suggested a *general* deficit in response to affective cues in depression (Bylsma et al., 2008).

Of note, however, the impact of Pavlovian bias is dependent on individuals having obtained the learned association. If they do not know that a cue leads to reward, then they will not be driven to approach it. As such, the impact of these sorts of biases may change over time. They will have minimal impact at the start of the process before the cue–outcome contingencies have been aquired. Reduced Pavlovian influence in depressed patients could therefore plausibly reflect *delayed* instrumental learning in some cases (despite eventual intact learning, given enough trials). Notably, reduced Pavlovian influence in depressed individuals may differ from what is found in those with increased clinical anxiety symptoms who demonstrate *increased*reliance on Pavlovian avoidance (and not approach) biases (Mkrtchian et al., 2017). Existing work is on this topic is limited, so further exploration of the interaction between Pavlovian bias and instrumental learning in major depressive disorder (MDD) would be worthwhile.

### Choice Variability (Temperature)

Returning to the softmax choice function (Equation 1), we can add another parameter, which provides a modulatory impact on choice. This parameter is referred to in different ways across the literature, but one common description is *temperature*, with greater temperature referring to greater response variability:

$${ActionProbability}_{\left\{s_{t}\}(a\right)}=\left[\frac{\text{exp}\left(\frac{{Value}_{\left\{s_{t}\}(a\right)}}{Temperature}\right)}{\sum_{b=1}^{n}\text{exp}\left(\frac{{Value}_{\left\{s_{t}\}(b\right)}}{Temperature}\right)}\right].$$ (4)

In principle, it should be possible to distinguish changes in temperature-driven choice variability from the effect of value in the learning model, but in practice, it can be difficult to do so because of the ambiguity surrounding behavioral indicators of exploration. Specifically, choice variability could result from a *deliberate* exploration of the presented options or simply from a failure to learn or otherwise express the true value of a given choice. Equation 4 reveals the problem explicitly: Value and temperature are directly proportional such that increases in value can simply be counteracted by increases in temperature (or vice versa).

Increased reliance on the temperature parameter has been used to explain performance on tasks in which depression is linked to increased exploration (Huys et al., 2012; Kunisato et al., 2012). Reinforcement learning–based analyses have argued that increased exploration drives response switching on RL tasks (Blanco et al., 2013), probabilistic reversal learning (Dombrovski, Szanto, Clark, Reynolds, & Siegle, 2013; Murphy et al., 2003; Taylor Tavares et al., 2008), and IGTs (Must et al., 2006). However, in many of these designs, it is also possible to explain behavior in terms of increased temperature. This, in turn, may be due to impaired task acquisition but could also be due to unmodeled decision-making strategies (e.g., the win–stay, lose–shift process described in section “Learning Rate”).

Perhaps the clearest evidence for the value–temperature trade-off comes from the probabilistic reward task (Pizzagalli, Jahn, & O’Shea, 2005), in which individuals are asked to discriminate between two lengths of lines presented on a screen. One of the responses has a greater probability of reinforcement following an accurate response, so that, for example, the accurate selection of the long line is three times more likely to be rewarded than the accurate selection of the short line. By providing asymmetric reward for one option, a reward-related bias is introduced in healthy individuals who normatively select the rewarded option if unsure (Huys et al., 2013). Individuals with high levels of anhedonia generally fail to show such a bias (Huys et al., 2013). Critically, a meta-analysis of this task using a computational analysis of performance identified increased temperature as the key parameter driving these effects in anhedonia (Huys et al., 2013). In other words, anhedonia led to noisier, more variable choices. As far as we are aware, the only existing study in which temperature was shown to be *reduced* by major depression/anhedonia (i.e., the opposite effect) was a study of the probabilistic selection task referred to in Table 1 (Chase, Frank et al., 2010). However, a reduced overall learning rate associated with anhedonia was also observed in this study, and it is generally difficult to dissociate estimation of temperature from overall performance or learning rate estimation (Daw, 2011). In summary, therefore, it seems plausible that anhedonia may be associated with increased temperature.

#### Simulation showing the importance of temperature in decision-making models

Looking at the softmax choice equation (Equation 4), increasing the value of the chosen option can be directly counteracted by increasing the temperature parameter, such that these parameters are now necessarily “underdetermined” or, more informally, “two sides of the same coin.” This point is emphasized by Huys and colleagues (2013) in their analysis of the probabilistic reward task and is valuable for two reasons. First, it increases focus on aspects of paradigm design and model fitting that might obscure identification of choice variability (or be obscured by it). Second, it has implications for sample size and statistical power: Specifically, choice variability, whether well specified or controlled by a particular task, or not, adds noise and provides an impediment to accurately identifying other modeled parameters. This could potentially affect the estimation of model parameters for many choice-oriented tasks across a variety of cognitive domains.

We can illustrate this interaction between value and temperature through simulation. Here we considered economic decision making under uncertainty (Figure 1C) rather than RL, because temperature is more transparently estimated in the absence of trial-by-trial learning (Daw, 2011). Critically, this simulation assumes that values and associated probabilities of outcomes are explicitly stated so that changes in decisions are not confounded with differences in learning the values:

$$Utility=Probability\cdot (EV^{EiskAversion}).$$ (5)

Consider the following: A decision-making paradigm of 100 trials assesses risk preference by asking the participant to choose between a risky 50% chance of winning $1 (vs. $0) or a sure 100% chance of winning $0.50 (i.e., on average, the value of both options is the same). Preference is determined by the relative valuation of $0.50 relative to $1, for example, a curved utility function (Equation 5). If risk aversion is greater than 1, the risky option is preferred, and if it is less than 1, the safe option is preferred. However, we might also assume that choice is stochastic, controlled by a softmax function and an accompanying temperature parameter (Equation 4).

**Figure 2.**
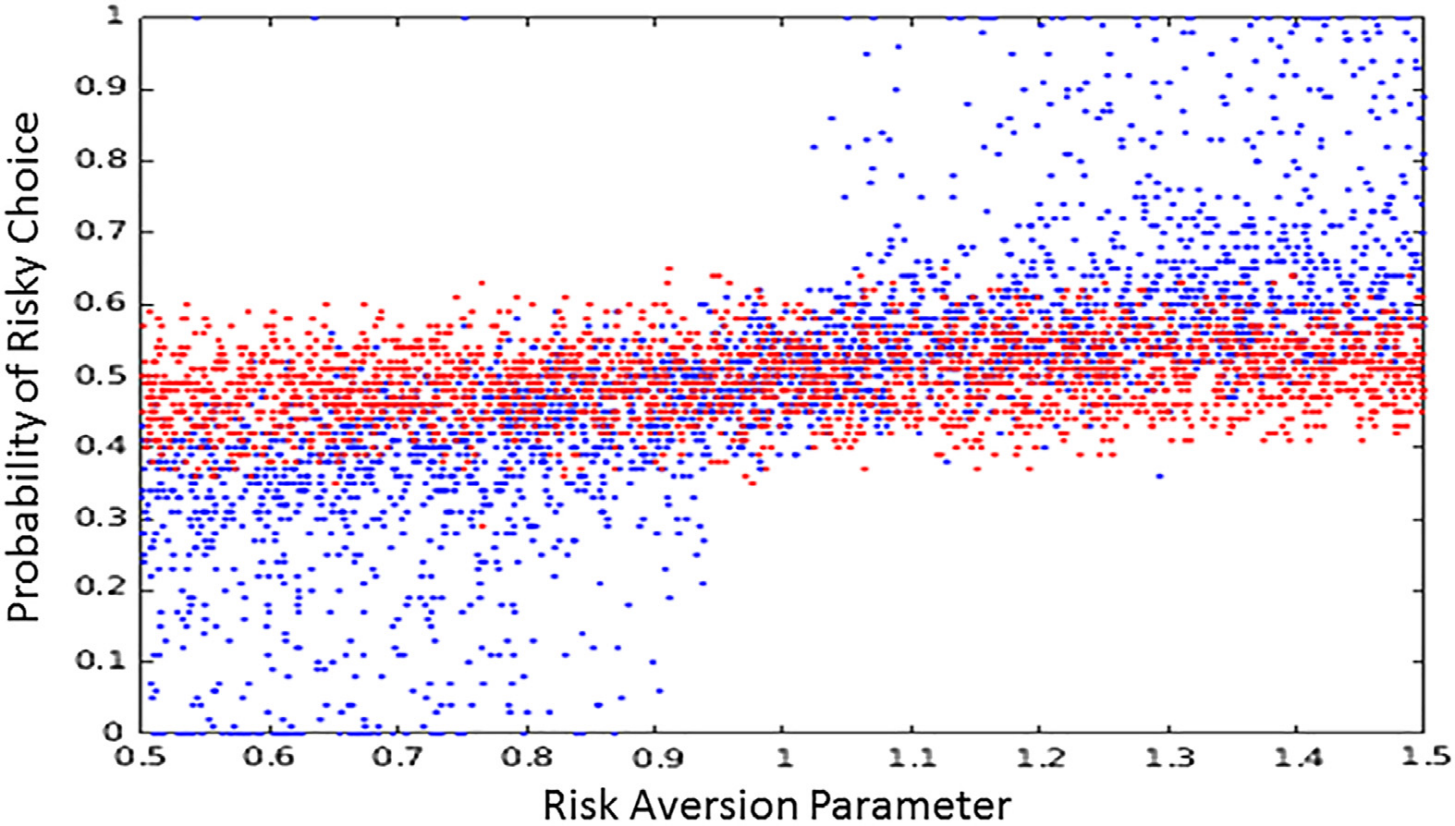
**Moderation of relationship between the risk aversion parameter (see Equation 5) and risk preference by temperature.** High temperatures are red; low temperatures are blue. A low score on the risk aversion parameter amplifies the utility of small wins, leading to risk aversion, but this is only clearly manifest in behavior if the temperature is low. Likewise, a high score reduces the utility of small wins, leading to risk seeking, but again, only if the temperature is low.

We conducted 10,000 simulations of this scenario, allowing temperature to vary between 0 and 2 and the curvature (risk aversion) to vary between 0.5 and 1.5.^1^ A multiple regression model was then fit to the resulting data, with risk preference (the proportion of risky options selected) being predicted by temperature, risk aversion, and their interaction. As expected, the curvature/risk aversion parameter (*t* = 67.96) was a highly significant predictor of risky choice, and temperature on its own had little effect (*t* = −1.87). Critically, however, their *interaction* was highly significant (*t* = −62.99). This interaction reflects the crucial influence of the temperature parameter, controlling the effect of the mapping between the key construct of interest (the valuation function) and the dependent measure (risk preference; see Figure 1C). This simulation demonstrates how temperature can play a crucial moderating role, with increases in temperature diminishing the influence of a given manipulation (in this case, risk aversion). Specifically, as can be seen by the red dots in Figure 2, at high temperatures, the probability of a risky choice is essentially random (50%) regardless of the level of risk aversion, and the curvature evident in the low-temperature blue dots is absent.

Ultimately, choice variability can have a major impact on observed behavior, and failing to account for it may lead to erroneous inferences regarding the influence of an experimental manipulation. For instance, reduced temperature might lead to a pattern of risky choice in healthy individuals, while high temperature would lead to (relatively) more random responding in a patient group. A pattern of noisier decisions could therefore be incorrectly taken as evidence of reduced risk taking in the patient group. In fact, the consequences are much broader than the present example: High temperature would make any other parameter within the same model harder to estimate, regardless of the paradigm or direction of between-group effects.

#### A role for temperature in accounting for the influence of effort manipulations?

With the preceding simulation in mind, enhanced temperature-driven choice variability might plausibly help explain conflicting findings within the literature. For example, there are ambiguities in data obtained using effort-based paradigms (e.g., Clery-Melin et al., 2011; Sherdell et al., 2012; Treadway et al., 2012) in which subjects are required to complete effortful tasks (e.g., press a button multiple times quickly or squeeze something with high pressure). On one hand, Treadway and colleagues (2012) found that anhedonic depressed participants were less likely overall to select high-effort option choices with a favorable expected value than healthy control subjects (see also Hershenberg et al., 2016). This would be consistent with reduced sensitivity to reward, given that effort-related costs are matched between the samples. On the other hand, Clery-Melin et al. (2011) showed that, unlike controls, MDD patients did not increase their grip force to obtain rewards of greater value but rather showed similar (mean) output across all rewards. However, this study *also* observed that effort could be enhanced in response to arousing stimuli in MDD patients but not in controls. In other words, some stimuli *do* have the capacity to elicit increases in effort in MDD patients, and effort expenditure is not reduced overall; rather, the increase in reward magnitude on this task is specifically ineffective at achieving increases in effort. This goes back to the point that most human research tends to employ forced-choice paradigms in which behavior is described by a choice between two options (i.e., 2AFC). If both options have low motivational properties overall, this would lead to low expected value and hence low overall action probability (e.g., Equation 4). In other words, if neither option is of much interest to the anhedonic participant, then he or she will respond more randomly—likely resulting in a high estimate of the temperature parameter. We should stress that this does not necessarily negate the role of reward sensitivity nor effort costs in the observed effort task effects (Treadway et al., 2012); rather, this highlights the broader potential of computational approaches in clarifying observed discrepancies.

### Anhedonia Reduces Drift Rate but No Other Parameters in Drift Diffusion Models?

A smaller number of studies have explored the effect of the drift diffusion model in depression. In a study using the flanker task—where subjects have to identify the orientation of an arrow in the face of congruent and incongruent proceeding distractors—depressed individuals were slower but more accurate on incongruent trials. Modeling showed that this was driven by reduced prepotent and executive drift rates across both congruent error and correct incongruent trials (Dillon et al., 2015) in depressed individuals compared to controls. This replicated an effect of reduced drift rate in depressed patients on a signal (color ratio) detection task (Vallesi, Canalaz, Balestrieri, & Brambilla, 2015) and prior work with the flanker task (Pe, Vandekerckhove, & Kuppens, 2013). Critically, drift rate correlated negatively with overall depressive symptoms (Pe et al., 2013; Vallesi et al., 2015) and with anhedonia specifically (Dillon et al., 2015). In none of the studies was depression associated with increased nondecision time, arguing against a generic psychomotor slowing effect; decision threshold (boundaries), arguing against speed–accuracy trade-offs (Dillon et al., 2015); or starting point, arguing against asymmetrical differences in boundaries.

However, it should be noted that there is some concern that the diffusion model can be overspecified, especially when trial numbers are limited and the study is not designed to detect more subtle effects (van Ravenzwaaij, Donkin, & Vandekerckhove, 2017). Indeed, very basic versions of the model can outperform more complex versions in simulations (van Ravenzwaaij et al., 2017). This is because more complex models can lead to overfitting, especially when the number of trials recorded is limited, which in turn reduces the power to detect group differences in core parameters (e.g., decision boundaries; van Ravenzwaaij et al., 2017). Comprehensive model comparison is needed in future research to be confident that effects of depression are in fact restricted to drift rate.

## NEUROIMAGING OF THE ANHEDONIC PHENOTYPE

### Altered Reward-Related Striatal Reactivity in Major Depressive Disorder

The learning and decision-making processes described can be integrated with neuroimaging in an attempt to map model parameters onto the underlying neuronal hardware and bridge the symptomatic, computational, and neurobiological levels. Release of dopamine in the striatum has long been linked to RL models (Kishida et al., 2016; Lohrenz, Kishida, & Montague, 2016; Pessiglione, Seymour, Flandin, Dolan, & Frith, 2006), so a large body of work has focused on the role of the striatum in anhedonia and depression. Before discussing explicitly computational work, we first highlight noncomputational work implicating the ventral striatum (VS) in anhedonia and depression.

A widely used paradigm for assessing reward and motivational processes in psychiatric populations is the *monetary incentive delay* paradigm (MID; Knutson, Fong, Adams, Varner, & Hommer, 2001; Knutson & Heinz, 2015). Although there are a variety of task versions, the general structure involves an expectancy cue that informs the participant about the amount of a reward he or she will receive if he or she makes a fast response. In general, the task is designed such that it is possible to separately model neural responses to (a) anticipation of reward and (b) receipt of rewarded outcomes.

**Table 2.** Exploring reward processing in the striatum

**Study**	**Groups**	**Outcome magnitude**	**Probability (%)**	**Response contingent**	**Task length**	**Striatum differences**	**Reported null findings**
Hagele et al. (2015)	AUD, SZ, MDD, BD (manic), ADHD, HC	±€0.1, €0.6, €3	67	Yes	2 × 72 trials	Right VS: Increasing depression severity reduces reward anticipation vs. neutral	
Stoy et al. (2012)	MDD (before and during treatment), HC	€0.1, €0.6, €3	67	Yes	2 × 72 trials	VS: HC > MDD, reward and loss anticipation vs. neutral—partially recovers after treatment	
Knutson, Bhanji, Cooney, Atlas, & Gotlib (2008)	Unmedicated MDD, HC	± $0.1, $0.2, $1, $5	67	Yes (individually calibrated RT threshold)	2 × 90 trials	Putamen: HC > MDD, reward outcome vs. neutral	VS: Reward anticipation
Admon et al. (2015)	MDD, HC	Variable: mean +$2.15, –$2	50	No; instructed	5 × 24 trials	Caudate: HC > MDD, reward and loss outcomes vs. neutral	
Wacker, Dillon, & Pizzagalli (2009)	Healthy individuals varying in anhedonic symptoms	Variable: mean +$2.15, –$2	50	No; instructed	5 × 24 trials	VS: Increasing anhedonia reduces reward outcome vs. neutral	VS: Reward anticipation
Pizzagalli et al. (2009)	MDD, HC	Variable: mean +$2.15, –$2	50	No; instructed	5 × 24 trials	Putamen: HC > MDD, reward anticipation vs. neutral; Caudate/VS: HC > MDD, reward outcome vs. neutral;	VS: Reward anticipation
Smoski, Rittenberg, & Dichter (2011)	MDD, HC	Money (+$1), IAPS pictures	67	Yes	2 × 2 × 40 trials	Putamen: Anticipation Group × Reward Type interaction	Widespread anticipation-related activation; little outcome-related activation
Arrondo et al. (2015)	MDD, SZ, HC	High (£1), low (£0.01)	70 high win, 30 low win	No; instructed	30 win, 30 neutral trials	VS: HC > MDD/SZ, reward anticipation; relationship of VS anticipation activation with anhedonia in SZ, not MDD	
Dichter, Kozink, McClernon, & Smoski (2012)	Remitted MDD, HC	+$1 for wins	67	Yes	20 potential win, 20 neutral	Caudate: remitted MDD > HC, reward anticipation	
Mori et al. (2016)	Students with/without subthreshold depression	±¥0, ¥20, ¥100, ¥500	N.S.	N.S.	40 gain, 40 loss, 10 neutral	Differences not within striatum	VS: Reward anticipation
Misaki, Suzuki, Savitz, Drevets, & Bodurka (2016)	MDD, HC	±$0.2, $1	66	Yes (individually calibrated RT threshold)	15 high win, 15 low win, 15 neutral, 15 high loss, 15 low loss	Left VS: HC > MDD during high win anticipation	No differences seen at low reward anticipation in left VS or low/high anticipation on right VS; no outcome- locked differences but overall activations not strong
Ubl et al. (2015)	Remitted MDD, HC	High (±€2), low (±€0.2) wins and losses	50% (approx.)	Yes (individually calibrated RT threshold)	N.S.	Differences not within striatum	
Stringaris et al. (2015)	Clinical, subthreshold depression, HC (adolescent)	10, 2, 0 points	66 (approx.)	Yes (individually calibrated RT threshold)	66 trials	VS: HC > clinical/ subthreshold depression, reward anticipation; reduced VS activation to reward anticipation also predicted transition to depression at 2-year follow-up and was related to symptoms of anhedonia. VS: Subthreshold depression > HC, positive outcomes Subthreshold depression and anhedonia > HC, negative outcomes	

*Note.* Table summarizing design and findings of studies of MDD or other depression-related cohorts that employed a reward-based version of the MID task. ADHD = attention-deficit hyperactivity disorder. AUD = alcohol use disorder. BD = bipolar disorder. N.S. = not stated. RT = reaction time. The contents of the table represent all the studies we were able to find using systematic searches for monetary incentive delay fMRI studies. A recent study of Admon et al. (2017) was not included, as it was focused on a dopaminergic drug manipulation, but it also found significant group (control > MDD) differences in the VS coupled to outcomes in the placebo condition.

We identified 13 studies in the literature using this task that are of relevance to the present discussion (Table 2). The task elicits quite reliable activation within the striatum in healthy individuals (Wu, Samanez-Larkin, Katovich, & Knutson, 2014), but there are inconsistencies as to when this activation occurs. While most studies reveal reduced activity in depression and anhedonia, they are coupled variably to anticipation or outcome and to different regions within the striatum. It is often less explicitly articulated (but see Knutson, Bhanji, Cooney, Atlas, & Gotlib, 2008) that the presence of null results—no difference between patients and controls—opens the potential for robust VS *activations* in the MDD group. Thus there is an obvious parallel with behavioral studies reviewed earlier: While deficits are regularly observed, the overall picture is not one of global hyporesponsivity to rewards. In addition, although there is a theme that less severely depressed individuals are less likely to show deficits (Hagele et al., 2015), Table 2 includes some studies with relatively severe patients that report null findings (Knutson et al., 2008).

It should be also noted that some studies, one in healthy adults (Schlagenhauf et al., 2013) and another in a geriatric depressed population (Dombrovski et al., 2015), have demonstrated correlations between VS activation and generic measures of cognitive function (IQ and self-reported executive function, respectively). These findings provide a complication in that cognitive dysfunction and anhedonic symptoms may be correlated and confounded. It is not always possible to correct for these associations within a given analysis. Moreover, the limitations of reverse inference are relevant in interpreting striatal activations coupled to reward events (Poldrack, 2011). Specifically, a reduction in striatal response, frequently observed in the absence of behavioral or symptomatic effects, is often interpreted as reduced reward sensitivity. This is clearly an oversimplification, as many studies have shown that striatal activation can be elicited by a wide range of behaviors and stimuli, including, for instance, punishment (Robinson, Overstreet, Charney, Vytal, & Grillon, 2013). Nevertheless, we interpret the evidence in Table 2 as broadly supporting a role of attenuated VS response to reward in anhedonia.

### Integrating Computational Approaches and Brain Imaging

The work reviewed in Table 2 nevertheless shows considerable heterogeneity. As argued earlier, computational models can help isolate sources of this variability. The integration of computational and fMRI methods is sometimes referred to as *model-based* analysis and represents a departure from the typical approach to modeling stimuli in neuroimaging that assumes that the eliciting stimulus is essentially equivalent on every trial. Specifically, computational models enable the exploration of parameters that vary on a trial-by-trial basis within subjects. Parameters such as temperature, explored in the section “Simulation Showing the Importance of Temperature in Decision-Making Models,” are generally fixed for individual subjects (i.e., they differ between subjects but do not change on a trial-by-trial basis), but these fixed values can be combined with the pattern of rewards and punishments received by the individual to calculate trial-by-trial regressors like PEs or value. These regressors can then be used as param etric modulators of functional magnetic resonance imaging acquisition outputs over time to determine their neural correlates. Specifically, PEs at a given time represent deviations from expected values (see also Equation 2).

$${Prediction\;Error}_{\left\{t\right\}}=(Sensitivity\cdot {outcome}_{\left\{t\right\}})-{Value}_{\left\{s_{t}\}(a_{t}\right)},$$ (6)

where the expected values are often updated according to a learning rate:

$${Value}_{\left\{s_{t}\}(a_{t}\right)}⇐({Value}_{\left\{s_{t}\}(a_{t}\right)})+\left(LearningRate\cdot {PredictionError}_{\left\{t\right\}}\right).$$ (7)

Previous studies, particularly by Steele and colleagues (2007), have successfully used these or similar models to describe blood oxygenation level–dependent signal changes in the VS, finding that activation coupled to appetitive PEs is altered in major depression (Gradin et al., 2011; Kumar et al., 2008). Other groups have made compatible observations (e.g., Robinson, Cools, Carlisi, Sahakian, & Drevets, 2012; Ubl et al., 2015) such that there is, at present, some evidence of attenuated outcome-locked reward PEs in major depression.

#### Reinforcement learning–linked striatal response locked to cue or outcome?

The studies in Table 2 generally reported changes to striatal activation locked to outcome *or* cue. However, the exact timing of the signal has critical implications for our understanding of the underlying deficit. Reduced anticipatory responses but intact outcome-locked responses might suggest a failure to learn about rewards (i.e., if a person has not learned that a cue predicts reward, the individual is not going to anticipate it) but an intact response to the rewards when received. The opposite pattern—reduced outcome but intact anticipation—can be harder to explain in the computational RL framework. Specifically, if outcome value is diminished (i.e., an individual cares less about rewards when they are received), then it is unclear how the individual would learn about the value of those rewards to develop an intact anticipation response. In other words, within RL, an intact response to value at outcome is a crucial step in the pathway to develop intact anticipation of that value. How, therefore, do we account for the outcome-linked effects reviewed in Table 2?

One potential explanation for outcome-linked effects is that there is faster learning or habituation in one group, such that early on in a paradigm, a participant might show intact outcome responses that enable learning, but then this outcome response (but not the anticipation response) declines over time, leaving the anticipatory response in place. It may also potentially be explained within a framework that posits separate systems for anticipation/preparation and consummation, such as wanting versus liking (Berridge & Robinson, 2003), sign tracking versus goal tracking (Flagel, Watson, Robinson, & Akil, 2007), or even anticipatory versus consummatory anhedonia (Argyropoulos & Nutt, 2013).

Another way to account for anticipation versus outcome effects is to account for them explicitly within the same model. For example, Kumar and colleagues (2008) fitted a RL model to reward-related VS activations, observing reward-related hypoactivation in MDD, but they modeled cue and outcome stages within the same framework. Specifically, they employed a modified temporal difference (TD) model (Sutton & Barto, 1998): a real-time development of RL in which reward PEs are computed continuously *within* a trial, with reference to expected values. This model has outcome PEs (like Equation 6) but also represents PEs during *anticipation*. Specifically, cues that predict rewards can also elicit their own reward PEs if their presence is uncertain but (more or less) reliably predicts reward. Thus the TD PE signal represents deviations from the expected upcoming reward, whether signaled by cues or experienced directly at the outcome. Kumar and colleagues’ (2008) finding that TD-coupled striatal responses were reduced in MDD compared to controls represents a development of the traditional, independent modeling of cue- and outcome-locked activation, as described in Table 2, toward a unified account in terms of a TD-derived PE signal.

A final way to account for outcome effects is to examine statistical relationships *between* anticipation- and outcome-locked striatal activations. Across two MDD cohorts using a reward-based guessing task, cue-locked reward expectancy and outcome-locked PE-related activations appeared similar across patients and controls, but the *relationship between them* differed (Chase et al., 2013; Greenberg et al., 2015). Specifically, on one hand, HC showed a negative relationship between expectancy and outcome, suggesting a different rate of learning-induced transmission from outcome to cue-related activity. On the other hand, individuals with MDD did not show this relationship, despite showing similar overall magnitude of reward anticipation- and outcome-locked responses. When examining this effect at the whole-brain level using individual differences in anhedonia as a moderator (Greenberg et al., 2015), the largest effect size was found very close to a meta-analytically determined anterior caudate region of the VS (Zhang, Chang, Guo, Zhang, & Wang, 2013).

Although the reason for this anticipation–outcome correlation finding is unclear, it provides a novel interpretation of the mixed findings in Table 2 and of unified accounts, such as that of Kumar and colleagues (2008). Specifically, current RL models may not fit to VS activation in MDD owing to the presence of an unmodeled relationship between outcome and cue responses. In other words, attenuated reward-related activation in MDD might simply reflect an inadequate fit of the general linear model rather than hypoactivation per se (Xu, 2015). Critically, this provides a direct parallel with our behavioral simulation (see the section “Simulation Showing the Importance of Temperature in Decision-Making Models”), in which apparent reductions in reward response can actually be driven by poor model fit rather than meaningful parameter differences. Future work, ideally comprising more complex models alongside principled model comparison, is necessary to clarify this.

## FIVE QUESTIONS FOR FUTURE RESEARCH

The evidence reviewed earlier reveals a promising start. Notably, the computational approach may provide potential ways of reconciling apparently contradictory findings about the role of reward processing and the VS in anhedonia. However, as is also clear from the preceding simulations, the computational approach adds its own set of complications and assumptions that require further evaluation. In this final section, we outline five unresolved questions, which we believe will prove fruitful for future research.

### What Is the Role of Temperature?

As discussed, it may well be that some decision-making differences in anhedonia are down to noisier decisions captured by the temperature parameter, but a major empirical challenge for studying the temperature parameter is that it is difficult to separate from value (see also Huys et al., 2013). Given that these two, supposedly distinct, constructs are inextricably linked within the choice rule (e.g., the softmax in Equation 4), it may not be possible to disambiguate them cleanly within one single paradigm. Moreover, although other choice rules for 2AFC paradigms might be considered, they often show very similar properties and a similar underlying mathematical form (Bogacz, Brown, Moehlis, Holmes, & Cohen, 2006; Yuille & Gaiger, 2003).

We therefore identify four potential strategies for resolving the role of temperature. First, if noisy, temperature-driven choices are a critical component of anhedonia, then they should be seen across different paradigms and task contingencies. If high choice variability were not also seen on other, conceptually distinct paradigms in the same individuals, then it might be possible to build a case that the effects on an effort task (for instance) are not driven by temperature differences. Second, it may be possible to manipulate outcome uncertainty to promote or reduce noisy decisions (Le Pelley, Suret, & Beesley, 2009), while keeping value consistent (and vice versa). Alternatively, forms of directed exploration have been proposed (Frank, Doll, Oas-Terpstra, & Moreno, 2009; Wilson, Geana, White, Ludvig, & Cohen, 2014), which might exist independently of a more passive, disinterested choice variability and be revealed on certain kinds of paradigms. The temperature parameter as realized within the softmax algorithm (Equation 4) is theoretically silent on this difference, and it may be that isolating these two components will provide more specificity into the underlying alteration in individuals with MDD. Together, these experimental approaches might enable the identification of exploration-related phenomena, which can be modeled independently of option value.

Third, it is worth considering whether a participant whose data provide evidence of high choice variability is just poorly fit by the model employed. Temperature could simply reflect the “residual” behavior that is not explained by the model itself. Thus other potential models of behavior might be explored that would better account for observed performance to clarify whether increased temperature, rather than just a poorly fitting model, is indeed the driving factor. Full comparison of a wide range of models, followed by clear simulations that reiterate nonmodeled behavioral patterns, can help determine the extent of this issue (Palminteri et al., 2017).

Finally, following from the preceding, large sample sizes can also provide benefits to model comparison and fitting. They can provide a clearer picture of parameter distributions and assist in their estimation, while nonnormal or discontinuous distribution of data (e.g., Chase et al., 2017; Chung et al., 2017) may be more readily identified. In addition, deriving more precise predictions from previous studies and/or via direct replication may facilitate Bayesian parameter estimation (Gershman, 2016).

### Can We Disentangle Responses to Outcome Versus Cue?

Adopting a reinforcement learning–based approach to modeling and interpreting ventral striatal activation may help to explain differences between findings arising from reward paradigms of different design or contingencies (e.g., see Table 2), without needing to suggest separate mechanisms for cue- and outcome-locked activation. For instance, some paradigms may employ relatively sparse reinforcement, that is, a weak relationship between predictive cues and rewards (e.g., Segarra et al., 2015), and thus show mostly low cue-locked reward anticipation. In this case, outcome-locked activation would be expected to carry a mostly PE-related signal, but it may also vary between subjects or groups in terms of outcome sensitivity, but not in terms of learning rate. In other words, a patient versus control group difference in a sparse reinforcement contingency is more likely to be driven by differences in outcome value or PE than by differences in learning rate. By contrast, in paradigms in which rewards are more frequent, and preceding cues can be effective predictors of the value of the outcome, both outcome- and anticipation-locked activation might show variability related to individual differences in both learning rate and outcome value. Thus, in this case, a group difference related to learning rate might be revealed in a way that would not be apparent in a sparse reinforcement design. Direct exploration of the impact of sparse versus frequent rewards in anhedonic individuals may help shed some light on this issue.

However, there are areas where the explanatory capability of a simple RL model may become limited. Importantly, for example, RL in neuroimaging has generally been applied to describe phasic, spiking responses of midbrain dopamine (DA) neurons (Schultz, Dayan, & Montague, 1997), as opposed to tonic DA release. While modification of the RL framework has been suggested to account for tonic DA fluctuations (Daw, Kakade, & Dayan, 2002; Niv, Daw, Joel, & Dayan, 2007), other evidence suggests that DA release at different timescales can be integrated into a single signal (Hamid et al., 2016). The operation of motivational systems at different timescales may be relevant for MDD, with respect to both behavioral evidence (Dillon et al., 2015) and neural responses to the cue and to the outcome. In particular, several studies have suggested that *sustained* rather than phasic responses to reward can be associated with MDD (Admon & Pizzagalli, 2015) and response to treatment (Heller et al., 2013). Thus building models that distinguish between the phasic and sustained responses to reward is likely to be an important future direction and may contribute to resolving some of the existing discrepancies with respect to cue- and outcome-locked activations.

### What Is the Role of Patient Heterogeneity?

Much of the logic behind the present work is based on the idea that patients with MDD will show high levels of anhedonia, which might drive the reward-related deficits observed. However, although it is true that anhedonia is a core clinical feature of MDD, a diagnosis of MDD may be given on the basis of heightened negative mood, despite normal hedonic tone. A radical consequence of this heterogeneity may be the inability to reproduce patterns of neuroimaging findings across cohorts (Muller et al., 2017). Although reward-based neuroimaging paradigms have been successful in identifying differences between patients with MDD and controls (Zhang et al., 2013), analyzing data as a function of continuous constructs may provide more rigorous tests by accounting for the heterogeneity within a given cohort. For example, using continuous measures, such as anhedonia questionnaire scores—as suggested by the research domain criteria framework (Kozak & Cuthbert, 2016)—may help resolve aspects of the conflicting data presented in Table 2. However, this endeavor will also benefit from more effective and validated measures of anhedonia (Rizvi et al., 2016). In this regard, computational methods may provide some assistance: A metric of approach motivation or reward sensitivity derived from a computational model of behavior could, for instance, be used as a between-subject continuous variable in such analyses.

Medication remains another important source of variability, one that can be difficult to control in patient populations (e.g., Hafeman, Chang, Garrett, Sanders, & Phillips, 2012). Potential solutions to this problem are to (a) examine the effect of medication in a control population on a comparable task (Kumar et al., 2008) or (b) examine unmedicated patients to compare with findings from medicated patients (Greenberg et al., 2015; Robinson et al., 2012). All of these possibilities are associated with inferential blind spots, but there should be no reason why the influence of medication could not be isolated eventually, as has been possible with Parkinson’s disease, for example (Cools, Barker, Sahakian, & Robbins, 2001).

### How Does Anxiety Interact With Anhedonia?

Related to the question of patient heterogeneity is comorbidity. Anhedonia is highly comorbid with anxiety. If anhedonia can be operationally characterized as reduced processing of rewards, the affective state of anxiety can be operationally characterized as increased processing of threats (Davis, Walker, Miles, & Grillon, 2010; Grillon, 2008; Robinson, Vytal, Cornwell, & Grillon, 2013). These are complementary and competing drives, and a full understanding of mood disorders will likely require integrating understandings of both.

Prior work using computational approaches to understand elevated aversive processing in anxiety is, however, extremely limited (but for a review, see Raymond, Steele, & Series, 2017). From a RL perspective, anxiety induced by threat of shock has been shown to *increase* a positively signed aversive PE signal to unexpected fearful face stimuli in the VS (Robinson, Overstreet, et al., 2013). This replicates animal work (Oleson, Gentry, Chioma, & Cheer, 2012) and is broadly consistent with a wide range of studies implicating the VS in aversive PEs under normal conditions (Delgado, Li, Schiller, & Phelps, 2008; Robinson, Frank, Sahakian, & Cools, 2010; Seymour, Daw, Dayan, Singer, & Dolan, 2007) but contrasts with the putative attenuated striatal response to reward in anhedonia reviewed earlier in this article. Similarly, while the earlier reviewed evidence did not suggest a role of learning rate differences in the manifestation of anhedonia, trait anxiety has been shown to be negatively associated with the ability to *modulate* learning rates (Browning et al., 2015) in response to environmental volatility. This could potentially result in aversive outcomes being experienced as less predictable and controllable than they are and thus help maintain the anxious state. Moreover, pathological anxiety has also been shown to *increase* rather than decrease reliance on Pavlovian avoidance parameters (Mkrtchian et al., 2017). Notably, in this particular study, the winning model had two separate Pavlovian parameters: one for approaching rewards and one for avoiding punishments. Anxiety disorders were associated with increased reliance on the avoidance parameter but not on the approach parameter. An interesting question for future work in anhedonia, therefore, is whether a similar, albeit attenuated bias exists for the approach parameter alone if it is modeled separately. Another study looking at models of economic decision making under uncertainty (Figure 1C) suggested that anxiety was associated with risk but not with loss aversion (Charpentier et al., 2016). Finally, there is evidence that high trait anxiety is associated with increased boundary separation and nondecision time on the drift diffusion model (White et al., 2010) rather than the reduced drift rate seen in anhedonia (although see Aylward, Hales, Robinson, & Robinson, 2017, for potential evidence of drift rate changes in anxiety disorders).

In sum, research into the computational parameters of anxiety, while in their infancy, reveals effects that might differ in important ways from those in anhedonia, and understanding how these interact in the clinical manifestation of mixed anxiety and depression is a key unresolved question.

### What Are the Clinical Implications?

The onset and causes of mood disorders remain puzzling (Kendler & Halberstadt, 2013). The efficacy of our dominant treatment strategies—pharmacotherapy and psychological therapy (NICE, 2014)—are unpredictable. The increasing empirical focus in the mood disorders literature on reinforcement processes has been accompanied by increasing acknowledgment of the antidepressant potential of dopaminergic agents (e.g., Fawcett et al., 2016; Racagni, Canonico, Ravizza, Pani, & Amore, 2004), which may act by influencing RL processes. As such, understanding individual differences in reward pathways, for example, using RL paradigms, may offer an effective way to stratify patients and predict treatment response. For example, SSRIs may show reduced efficacy for individuals with high levels of anhedonia and, hence, deficits in reward-related behavior (Vrieze et al., 2012), while there may be learning signatures that indicate that an individual will respond well to psychological intervention (see, e.g., Culver, Vervliet, & Craske, 2015). Future work integrating computational approaches with clinical trials may ultimately improve our ability to target treatments.

## OVERALL SUMMARY

The premise of the present review is that models of learning and choice can help illuminate the core symptoms of mood disorders. Indeed, we find some empirical support for the role of computational model parameters in explaining variability in behavioral and neural responses to obtaining rewards in anhedonia. Most notably, the reviewed data highlight—in contrast to the typical focus on reduced reward or enhanced punishment sensitivity—a potential role for attenuated response to Pavlovian biases, increased choice variability captured by temperature, and reduced drift rates in reaction times in driving anhedonia-linked behavioral variability. These changes may cumulatively manifest as the attenuated striatal response to rewards that are often observed in neuroimaging studies. Critically, however, the adoption of computational methods has brought to light factors that would have been difficult to identify previously. Our final five unresolved questions build on the insights from the reviewed findings: Resolving these questions will, we hope, take us a step closer to understanding the precise nature of the behavioral deficits underlying mood disorders and to the ultimate goal of improving outcomes for patients.

## AUTHOR CONTRIBUTIONS

Oliver J. Robinson and Henry W. Chase contributed equally to this article.

## FUNDING INFORMATION

Oliver J. Robinson is funded by a Medical Research Council Career Development Award (MR/K024280/1). Henry W. Chase is supported by NIMH award 1R21MH108421-01A1.

## Note

^1^ Code used in these simulations has been made available online at https://doi.org/10.6084/m9.figshare.5103322.v1 (Chase, 2017)

## References

[bib1] Admon R., Kaiser R. H., Dillon D. G., Beltzer M., Goer F., Olson D. P., … Pizzagalli D. A. (2017). Dopaminergic enhancement of striatal response to reward in major depression. American Journal of Psychiatry, 174, 378–386. 10.1176/appi.ajp.2016.16010111 27771973 10.1176/appi.ajp.2016.16010111 PMC5378658

[bib2] Admon R., Nickerson L. D., Dillon D. G., Holmes A. J., Bogdan R., Kumar P., … Pizzagalli D. A. (2015). Dissociable cortico-striatal connectivity abnormalities in major depression in response to monetary gains and penalties. Psychological Medicine, 45, 121–131. 10.1017/S0033291714001123 25055809 PMC4233014

[bib3] Admon R., & Pizzagalli D. A. (2015). Corticostriatal pathways contribute to the natural time course of positive mood. Nature Communications, 6, Article 10065 10.1038/ncomms10065 PMC4686763 26638823

[bib4] Argyropoulos S. V., & Nutt D. J. (2013). Anhedonia revisited: Is there a role for dopamine-targeting drugs for depression? Journal of Psychopharmacology, 27, 86–77. 10.1177/0269881113494104 23904408

[bib5] Arrondo G., Segarra N., Metastasio A., Ziauddeen H., Spencer J., Reinders N. R., … Murray G. K. (2015). Reduction in ventral striatal activity when anticipating a reward in depression and schizophrenia: A replicated cross-diagnostic finding. Frontiers in Psychology, 6, Article 1280 10.3389/fpsyg.2015.01280 26379600 PMC4549553

[bib6] Aylward J., Hales C., Robinson E., & Robinson O. J. (2017). Back-translating a rodent measure of negative bias into humans: The impact of induced anxiety and unmedicated mood and anxiety disorders. bioRxiv. 10.1101/143453 PMC7083556 30683161

[bib7] Beddington J., Cooper C. L., Field J., Goswami U., Huppert F. A., Jenkins R., … Thomas S. M. (2008). The mental wealth of nations. Nature, 455, 1057–1060. 10.1038/4551057a 18948946

[bib8] Beevers C. G., Worthy D. A., Gorlick M. A., Nix B., Chotibut T., & Todd Maddox W. (2013). Influence of depression symptoms on history-independent reward and punishment processing. Psychiatry Research, 207(1–2), 53–60. 10.1016/j.psychres.2012.09.054 23122555 PMC3566413

[bib9] Berridge K. C., & Robinson T. E. (2003). Parsing reward. Trends in Neuroscience, 26, 507–513. 10.1016/S0166-2236(03)00233-9 12948663

[bib10] Blanco N. J., Otto A. R., Maddox W. T., Beevers C. G., & Love B. C. (2013). The influence of depression symptoms on explor atory decision-making. Cognition, 129, 563–568. 10.1016/j.cognition.2013.08.018 24055832 PMC3809321

[bib11] Bogacz R., Brown E., Moehlis J., Holmes P., & Cohen J. D. (2006). The physics of optimal decision making: A formal analysis of models of performance in two-alternative forced-choice tasks. Psychological Review, 113, 700–765. 10.1037/0033-295X.113.4.700 17014301

[bib12] Box G. E. P. (1976). Science and statistics. Journal of the American Statistical Association, 71, 791–799. 10.2307/2286841

[bib13] Browning M., Behrens T. E., Jocham G., O’Reilly J. X., & Bishop S. J. (2015). Anxious individuals have difficulty learning the causal statistics of aversive environments. Nature Neuroscience, 18, 590–596. 10.1038/nn.3961 25730669 PMC4644067

[bib14] Bush R. R., & Mosteller F. (1955). Stochastic models for learning. Oxford, England: John Wiley.

[bib15] Bylsma L. M., Morris B. H., & Rottenberg J. (2008). A meta-analysis of emotional reactivity in major depressive disorder. Clinical Psychology Review, 28, 676–691. 10.1016/j.cpr.2007.10.001 18006196

[bib16] Cavanagh J. F., Bismark A. J., Frank M. J., & Allen J. J. (2011). Larger error signals in major depression are associated with better avoidance learning. Frontiers in Psychology, 2, Article 331 10.3389/fpsyg.2011.00331 22084638 PMC3210982

[bib17] Cavanagh J. F., Eisenberg I., Guitart-Masip M., Huys Q., & Frank M. J. (2013). Frontal theta overrides Pavlovian learning biases. Journal of Neuroscience, 33, 8541–8548. 23658191 10.1523/JNEUROSCI.5754-12.2013 PMC3707146

[bib18] Charpentier C. J., Aylward J., Roiser J. P., & Robinson O. J. (2016). Enhanced risk aversion, but not loss aversion, in unmedicated pathological anxiety. Biological Psychiatry, 81, 1014–1022. 10.1016/j.biopsych.2016.12.010 28126210 PMC5466268

[bib19] Chase H. (2017). Risk simulations. figshare. 10.6084/m9.figshare.5103322.v1. Retrieved: Nov 28, 2017.

[bib20] Chase H. W., Fournier J. C., Bertocci M. A., Greenberg T., Aslam H., Stiffler R., (2017). A pathway linking reward circuitry, impulsive sensation-seeking and risky decision-making in young adults: Identifying neural markers for new interventions. Translational Psychiatry, 7(4), e1096 10.1038/tp.2017.60 28418404 PMC5416701

[bib21] Chase H. W., Frank M. J., Michael A., Bullmore E. T., Sahakian B. J., & Robbins T. W. (2010). Approach and avoidance learning in patients with major depression and healthy controls: Relation to anhedonia. Psychological Medicine, 40, 433–440. 10.1017/S0033291709990468 19607754

[bib22] Chase H. W., Michael A., Bullmore E. T., Sahakian B. J., & Robbins T. W. (2010). Paradoxical enhancement of choice reaction time performance in patients with major depression. Journal of Psychopharmacology, 24, 471–479. 10.1177/0269881109104883 19406853

[bib23] Chase H. W., Nusslock R., Almeida J. R., Forbes E. E., LaBarbara E. J., & Phillips M. L. (2013). Dissociable patterns of abnormal frontal cortical activation during anticipation of an uncertain reward or loss in bipolar versus major depression. Bipolar Disorders, 15, 839–854. 10.1111/bdi.12132 24148027 PMC4065116

[bib24] Christianson J. P., Paul E. D., Irani M., Thompson B. M., Kubala K. H., Yirmiya R., (2008). The role of prior stressor controllability and the dorsal raphe nucleus in sucrose preference and social exploration. Behavioural Brain Research, 193, 87–93. 10.1016/j.bbr.2008.04.024 18554730 PMC2583404

[bib25] Chung D., Kadlec K., Aimone J. A., McCurry K., King-Casas B., & Chiu P. H. (2017). Valuation in major depression is intact and stable in a non-learning environment. Scientific Reports, 7, Article 44374 10.1038/srep44374 28281665 PMC5345037

[bib26] Clery-Melin M. L., Schmidt L., Lafargue G., Baup N., Fossati P., & Pessiglione M. (2011). Why don’t you try harder? An investigation of effort production in major depression. PLoS ONE, 6(8), e23178 10.1371/journal.pone.0023178 21853083 PMC3154289

[bib27] Collins A. G., Brown J. K., Gold J. M., Waltz J. A., & Frank M. J. (2014). Working memory contributions to reinforcement learning impairments in schizophrenia. Journal of Neuroscience, 34, 13747–13756. 10.1523/JNEUROSCI.0989-14.2014 25297101 PMC4188972

[bib28] Collins A. G., & Frank M. J. (2012). How much of reinforcement learning is working memory, not reinforcement learning? A behavioral, computational, and neurogenetic analysis. European Journal of Neuroscience, 35, 1024–1035. 10.1111/j.1460-9568.2011.07980.x 22487033 PMC3390186

[bib29] Cools R., Barker R. A., Sahakian B. J., & Robbins T. W. (2001). Enhanced or impaired cognitive function in Parkinson’s disease as a function of dopaminergic medication and task de mands. Cerebral Cortex, 11, 1136–1143. Retrieved from http://www.ncbi.nlm.nih.gov/entrez/query.fcgi?cmd=Retrieve&db=PubMed&dopt=Citation&list_uids=11709484 11709484 10.1093/cercor/11.12.1136

[bib30] Culver N. C., Vervliet B., & Craske M. G. (2015). Compound extinction using the Rescorla–Wagner model to maximize exposure therapy effects for anxiety disorders. Clincial Psychological Science, 3, 335–348.

[bib31] Davis M., Walker D. L., Miles L., & Grillon C. (2010). Phasic vs. sustained fear in rats and humans: Role of the extended amygdala in fear vs anxiety. Neuropsychopharmacology, 35, 105–135. 10.1038/npp.2009.109 19693004 PMC2795099

[bib32] Daw N. D. (2011). Trial-by-trial data analysis using computational models. In Delgado M. R., Phelps E. A., & Robbins T. W. (Eds.), Decision making, affect, and learning: Attention and performance XXIII (pp. 3–38). Oxford, England: Oxford University Press.

[bib33] Daw N. D., Kakade S., & Dayan P. (2002). Opponent interactions between serotonin and dopamine. Neural Networks, 15, 603–616. 10.1016/S0893-6080(02)00052-7 12371515

[bib34] DelDonno S. R., Weldon A. L., Crane N. A., Passarotti A. M., Pruitt P. J., Gabriel L. B., … Langenecker S. A. (2015). Affective personality predictors of disrupted reward learning and pursuit in major depressive disorder. Psychiatry Research, 230, 56–64. 10.1016/j.psychres.2015.08.011 26319737 PMC4601921

[bib35] Delgado M. R., Li J., Schiller D., & Phelps E. A. (2008). The role of the striatum in aversive learning and aversive prediction errors. Philosophical Transactions of the Royal Society, Series B, 363, 3787–3800. 10.1098/rstb.2008.0161 PMC2607367 18829426

[bib36] den Ouden H. E. M., Daw N. D., Fernandez G., Elshout J. A., Rijpkema M., Hoogman M., (2013). Dissociable effects of dopamine and serotonin on reversal learning. Neuron, 80, 1090–1100. 10.1016/j.neuron.2013.08.030 24267657

[bib37] Dichter G. S., Kozink R. V., McClernon F. J., & Smoski M. J. (2012). Remitted major depression is characterized by reward network hyperactivation during reward anticipation and hypoactivation during reward outcomes. Journal of Affective Disorders, 136, 1126–1134. 10.1016/j.jad.2011.09.048 22036801 PMC3272083

[bib38] Dillon D. G., Wiecki T., Pechtel P., Webb C., Goer F., Murray L., … Pizzagalli D. A. (2015). A computational analysis of flanker interference in depression. Psychological Medicine, 45, 2333–2344. 10.1017/S0033291715000276 25727375 PMC4499007

[bib39] Dombrovski A. Y., Szanto K., Clark L., Aizenstein H. J., Chase H. W., Reynolds C. F. III, & Siegle G. J. (2015). Corticostriatothalamic reward prediction error signals and executive control in late-life depression. Psychological Medicine, 45, 1413–1424. 10.1017/S0033291714002517 25319564 PMC4380546

[bib40] Dombrovski A. Y., Szanto K., Clark L., Reynolds C. F., & Siegle G. J. (2013). Reward signals, attempted suicide, and impulsiv ity in late-life depression. JAMA Psychiatry, 70, 1020–1030. 10.1001/jamapsychiatry.2013.75 PMC3859132 23925710

[bib41] Engelmann J. B., Meyer F., Fehr E., & Ruff C. C. (2015). Anticipatory anxiety disrupts neural valuation during risky choice. Journal of Neuroscience, 35, 3085–3099. 25698745 10.1523/JNEUROSCI.2880-14.2015 PMC6605597

[bib42] Fawcett J., Rush A. J., Vukelich J., Diaz S. H., Dunklee L., Romo P., … Escalona R. (2016). Clinical experience with high-dosage pramipexole in patients with treatment-resistant depressive epi sodes in unipolar and bipolar depression. American Journal of Psychiatry, 173, 107–111. 10.1176/appi.ajp.2015.15060788 26844792

[bib43] Flagel S. B., Watson S. J., Robinson T. E., & Akil H. (2007). Individual differences in the propensity to approach signals vs goals promote different adaptations in the dopamine system of rats. Psychopharmacology (Berlin), 191, 599–607. 10.1007/s00213-006-0535-8 16972103

[bib44] Frank M. J., Doll B. B., Oas-Terpstra J., & Moreno F. (2009). Prefrontal and striatal dopaminergic genes predict individual differences in exploration and exploitation. Nature Neuroscience, 12, 1062–1068. 10.1038/nn.2342 19620978 PMC3062477

[bib45] Franken I. H., Rassin E., & Muris P. (2007). The assessment of anhedonia in clinical and non-clinical populations: Further validation of the Snaith–Hamilton Pleasure Scale (SHAPS). Journal of Affective Disorders, 99(1–3), 83–89. Retrieved from http://www.ncbi.nlm.nih.gov/entrez/query.fcgi?cmd=Retrieve&db=PubMed&dopt=Citation&list_uids=16996138 16996138 10.1016/j.jad.2006.08.020

[bib46] Gershman S. J. (2016). Empirical priors for reinforcement learning models. Journal of Mathematical Psychology, 71, 1–6.

[bib47] Gradin V. B., Kumar P., Waiter G., Ahearn T., Stickle C., Milders M., … Steele J. D. (2011). Expected value and prediction error abnormalities in depression and schizophrenia. Brain, 134(Pt 6), 1751–1764. 10.1093/brain/awr059 21482548

[bib48] Gradin V. B., Perez A., MacFarlane J. A., Cavin I., Waiter G., Engelmann J., … Steele J. D. (2015). Abnormal brain responses to social fairness in depression: An fMRI study using the Ultimatum Game. Psychological Medicine, 45, 1241–1251. 10.1017/S0033291714002347 25277236

[bib49] Greenberg T., Chase H. W., Almeida J. R., Stiffler R., Zevallos C. R., Aslam H. A., … Phillips M. L. (2015). Moderation of the relationship between reward expectancy and prediction error-related ventral striatal reactivity by anhedonia in unmedicated major depressive disorder: Findings from the EMBARC study. American Journal of Psychiatry, 172, 881–891. 10.1176/appi.ajp.2015.14050594 26183698 PMC4858169

[bib50] Grillon C. (2008). Models and mechanisms of anxiety: Evidence from startle studies. Psychopharmacology, 199, 421–437. 10.1007/s00213-007-1019-1 18058089 PMC2711770

[bib51] Guitart-Masip M., Fuentemilla L., Bach D. R., Huys Q. J., Dayan P., Dolan R. J., (2011). Action dominates valence in antici patory representations in the human striatum and dopaminergic midbrain. Journal of Neuroscience, 31, 7867–7875. 10.1523/JNEUROSCI.6376-10.2011 21613500 PMC3109549

[bib52] Hafeman D. M., Chang K. D., Garrett A. S., Sanders E. M., & Phillips M. L. (2012). Effects of medication on neuroimaging findings in bipolar disorder: An updated review. Bipolar Disorders, 14, 375–410. 10.1111/j.1399-5618.2012.01023.x 22631621

[bib53] Hagele C., Schlagenhauf F., Rapp M., Sterzer P., Beck A., Bermpohl F., (2015). Dimensional psychiatry: Reward dysfunction and depressive mood across psychiatric disorders. Psychopharmacology (Berlin), 232, 331–341. 10.1007/s00213-014-3662-7 24973896 PMC4297301

[bib54] Hamid A. A., Pettibone J. R., Mabrouk O. S., Hetrick V. L., Schmidt R., Vander Weele C. M., … Berke J. D. (2016). Mesolimbic dopamine signals the value of work. Nature Neuroscience, 19, 117–126. 10.1038/nn.4173 26595651 PMC4696912

[bib55] Heller A. S., Johnstone T., Light S. N., Peterson M. J., Kolden G. G., Kalin N. H., & Davidson R. J. (2013). Relationships between changes in sustained fronto-striatal connectivity and positive affect in major depression resulting from antidepressant treatment. American Journal of Psychiatry, 170, 197–206. 10.1176/appi.ajp.2012.12010014 23223803 PMC3563751

[bib56] Hershenberg R., Satterthwaite T. D., Daldal A., Katchmar N., Moore T. M., Kable J. W., & Wolf D. H. (2016). Diminished effort on a progressive ratio task in both unipolar and bipolar depression. Journal of Affective Disorders, 196, 97–100. 10.1016/j.jad.2016.02.003 26919058 PMC4808384

[bib57] Herzallah M. M., Moustafa A. A., Natsheh J. Y., Abdellatif S. M., Taha M. B., Tayem Y. I., … Gluck M. A. (2013). Learning from negative feedback in patients with major depressive disorder is attenuated by SSRI antidepressants. Frontiers in Integrative Neuroscience, 7, Article 67 10.3389/fnint.2013.00067 24065894 PMC3779792

[bib58] Holland P. C. (2004). Relations between Pavlovian-instrumental transfer and reinforcer devaluation. Journal of Experimental Psychology: Animal Behavior Processes, 30, 104–117. 10.1037/0097-7403.30.2.104 15078120

[bib59] Huys Q. J., Eshel N., O’Nions E., Sheridan L., Dayan P., & Roiser J. P. (2012). Bonsai trees in your head: How the Pavlovian system sculpts goal-directed choices by pruning decision trees. PLoS Computational Biology, 8(3), e1002410 10.1371/journal.pcbi.1002410 22412360 PMC3297555

[bib60] Huys Q. J., Golzer M., Friedel E., Heinz A., Cools R., Dayan P., (2016). The specificity of Pavlovian regulation is associated with recovery from depression. Psychological Medicine, 46, 1027–1035. 10.1017/S0033291715002597 26841896 PMC4825095

[bib61] Huys Q. J., Maia T. V., & Frank M. J. (2016). Computational psy chiatry as a bridge from neuroscience to clinical applications. Nature Neuroscience, 19, 404–413. 10.1038/nn.4238 26906507 PMC5443409

[bib62] Huys Q. J., Pizzagalli D. A., Bogdan R., & Dayan P. (2013). Mapping anhedonia onto reinforcement learning: A behavioural meta-analysis. Biology of Mood & Anxiety Disorders, 3(1). 10.1186/2045-5380-3-12 PMC3701611 23782813

[bib63] Kendler K. S., & Halberstadt L. J. (2013). The road not taken: Life experiences in monozygotic twin pairs discordant for major depression. Molecular Psychiatry, 18, 975–984. 10.1038/mp.2012.55 22641178 PMC3523211

[bib64] Kishida K. T., Saez I., Lohrenz T., Witcher M. R., Laxton A. W., Tatter S. B., … Montague P. R. (2016). Subsecond dopamine fluctuations in human striatum encode superposed error signals about actual and counterfactual reward. Proceedings of the National Academy of Science of the United States of America, 113, 200–205. 10.1073/pnas.1513619112 PMC4711839 26598677

[bib65] Knox W. B., Otto A. R., Stone P., & Love B. C. (2011). The nature of belief-directed exploratory choice in human decision-making. Frontiers in Psychology, 2, Article 398 10.3389/fpsyg.2011.00398 22319503 PMC3269072

[bib66] Knutson B., Bhanji J. P., Cooney R. E., Atlas L. Y., & Gotlib I. H. (2008). Neural responses to monetary incentives in major depression. Biological Psychiatry, 63, 686–692. 10.1016/j.biopsych.2007.07.023 17916330 PMC2290738

[bib67] Knutson B., Fong G. W., Adams C. M., Varner J. L., & Hommer D. (2001). Dissociation of reward anticipation and outcome with event-related fMRI. Neuroreport, 12, 3683–3687. Retrieved from http://www.ncbi.nlm.nih.gov/entrez/query.fcgi?cmd=Retrieve&db=PubMed&dopt=Citation&list_uids=11726774 11726774 10.1097/00001756-200112040-00016

[bib68] Knutson B., & Heinz A. (2015). Probing psychiatric symptoms with the monetary incentive delay task. Biological Psychiatry, 77, 418–420. 10.1016/j.biopsych.2014.12.022 25645271

[bib69] Kozak M. J., & Cuthbert B. N. (2016). The nimh research domain criteria initiative: Background, issues, and pragmatics. Psychophysiology, 53, 286–297. 10.1111/psyp.12518 26877115

[bib70] Kumar P., Waiter G., Ahearn T., Milders M., Reid I., & Steele J. D. (2008). Abnormal temporal difference reward-learning signals in major depression. Brain, 131(Pt. 8), 2084–2093. 10.1093/brain/awn136 18579575

[bib71] Kunisato Y., Okamoto Y., Ueda K., Onoda K., Okada G., Yoshimura S., … Yamawaki S. (2012). Effects of depression on reward-based decision making and variability of action in probabilistic learning. Journal of Behavior Therapy & Experimental Psychiatry, 43, 1088–1094. 10.1016/j.jbtep.2012.05.007 22721601

[bib72] Lempert K. M., & Pizzagalli D. A. (2010). Delay discounting and future-directed thinking in anhedonic individuals. Journal of Behavior Therapy & Experimental Psychiatry, 41, 258–264. 10.1016/j.jbtep.2010.02.003 20219184 PMC2862767

[bib73] Le Pelley M. E., Suret M. B., & Beesley T. (2009). Learned predictiveness effects in humans: A function of learning, performance, or both? Journal of Experimental Psychology: Animal Behavior Processes, 35, 312–327. 10.1037/a0014315 19594278

[bib74] Lohrenz T., Kishida K. T., & Montague P. R. (2016). BOLD and its connection to dopamine release in human striatum: A cross-cohort comparison. Philosophical Transactions of the Royal Society of London, Series B, 371(1705). 10.1098/rstb.2015.0352 PMC5003854 27574306

[bib75] Maddox W. T., Gorlick M. A., Worthy D. A., & Beevers C. G. (2012). Depressive symptoms enhance loss-minimization, but attenuate gain-maximization in history-dependent decision-making. Cognition, 125, 118–124. 10.1016/j.cognition.2012.06.011 22801054 PMC3426306

[bib76] Martinez-Molina N., Mas-Herrero E., Rodriguez-Fornells A., Zatorre R. J., & Marco-Pallares J. (2016). Neural correlates of specific musical anhedonia. Proceedings of the National Academy of Sciences of the United States of America, 113, E7337–E7345. 10.1073/pnas.1611211113 27799544 PMC5135354

[bib77] Misaki M., Suzuki H., Savitz J., Drevets W. C., & Bodurka J. (2016). Individual variations in nucleus accumbens responses associated with major depressive disorder symptoms. Scientific Reports, 6, 21227 10.1038/srep21227 26880358 PMC4754797

[bib78] Mkrtchian A., Aylward J., Dayan P., Roiser J. P., & Robinson O. J. (2017). Modeling avoidance in mood and anxiety disorders using reinforcement learning. Biological Psychiatry, 82, 532–539. 10.1016/j.biopsych.2017.01.017 28343697 PMC5598542

[bib79] Montague P. R. (2012). The scylla and charybdis of neuroeconomic approaches to psychopathology. Biological Psychiatry, 72, 80–81. 10.1016/j.biopsych.2012.05.010 22727456 PMC4052708

[bib80] Montague P. R., Dolan R. J., Friston K. J., & Dayan P. (2012). Computational psychiatry. Trends in Cognitive Sciences, 16, 72–80. 10.1016/j.tics.2011.11.018 22177032 PMC3556822

[bib81] Mori A., Okamoto Y., Okada G., Takagaki K., Jinnin R., Takamura M., … Yamawaki S. (2016). Behavioral activation can normalize neural hypoactivation in subthreshold depression during a monetary incentive delay task. Journal of Affective Disorders, 189, 254–262. 10.1016/j.jad.2015.09.036 26454185

[bib82] Muller V. I., Cieslik E. C., Serbanescu I., Laird A. R., Fox P. T., & Eickhoff S. B. (2017). Altered brain activity in unipolar depression revisited: Meta-analyses of neuroimaging studies. JAMA Psychiatry, 74(1), 47–55. 10.1001/jamapsychiatry.2016.2783 27829086 PMC5293141

[bib83] Murphy F. C., Michael A., Robbins T. W., & Sahakian B. J. (2003). Neuropsychological impairment in patients with major depressive disorder: The effects of feedback on task performance. Psychological Medicine, 33, 455–467. Retrieved from http://www.ncbi.nlm.nih.gov/entrez/query.fcgi?cmd=Retrieve&db=PubMed&dopt=Citation&list_uids=12701666 12701666 10.1017/s0033291702007018

[bib84] Must A., Szabo Z., Bodi N., Szasz A., Janka Z., & Keri S. (2006). Sensitivity to reward and punishment and the prefrontal cortex in major depression. Journal of Affective Disorders, 90, 209–215. 10.1016/j.jad.2005.12.005 16412520

[bib85] Myers C. E., Sheynin J., Balsdon T., Luzardo A., Beck K. D., Hogarth L., … Moustafa A. A. (2016). Probabilistic reward- and punishment-based learning in opioid addiction: Experimental and computational data. Behavioural Brain Research, 296, 240–248. 10.1016/j.bbr.2015.09.018 26381438 PMC4734141

[bib86] NICE. (2014). Anxiety disorders NICE quality standard (Standard No. QS53). Retrieved from https://www.nice.org.uk/guidance/qs53.

[bib87] Niv Y., Daw N. D., Joel D., & Dayan P. (2007). Tonic dopamine: Opportunity costs and the control of response vigor. Psychopharmacology (Berlin), 191, 507–520. Retrieved from http://www.ncbi.nlm.nih.gov/entrez/query.fcgi?cmd=Retrieve&db=PubMed&dopt=Citation&list_uids=17031711 17031711 10.1007/s00213-006-0502-4

[bib88] Oleson E. B., Gentry R. N., Chioma V. C., & Cheer J. F. (2012). Subsecond dopamine release in the nucleus accumbens predicts conditioned punishment and its successful avoidance. Journal of Neuroscience, 32, 14804–14808. 10.1523/jneurosci.3087-12.2012 23077064 PMC3498047

[bib89] Palminteri S., Wyart V., & Koechlin E. (2017). The importance of falsification in computational cognitive modeling. Trends in Cognitive Sciences, 21, 425–433. 10.1016/j.tics.2017.03.011 28476348

[bib90] Pe M. L., Vandekerckhove J., & Kuppens P. (2013). A diffusion model account of the relationship between the emotional flanker task and rumination and depression. Emotion, 13, 739–747. 23527499 10.1037/a0031628

[bib91] Pechtel P., Dutra S. J., Goetz E. L., & Pizzagalli D. A. (2013). Blunted reward responsiveness in remitted depression. Journal of Psychiatric Research, 47, 1864–1869. 10.1016/j.jpsychires.2013.08.011 24064208 PMC3978009

[bib92] Pedersen M. L., Frank M. J., & Biele G. (2016). The drift diffusion model as the choice rule in reinforcement learning. Psychonomic Bulletin & Review, 24, 1234–1251. 10.3758/s13423-016-1199-y PMC5487295 27966103

[bib93] Pessiglione M., Seymour B., Flandin G., Dolan R. J., & Frith C. D. (2006). Dopamine-dependent prediction errors underpin reward-seeking behaviour in humans. Nature, 442, 1042–1045. Retrieved from http://www.ncbi.nlm.nih.gov/entrez/query.fcgi?cmd=Retrieve&db=PubMed&dopt=Citation&list_uids=16929307 16929307 10.1038/nature05051 PMC2636869

[bib94] Pizzagalli D. A. (2010). The “anhedonia paradox” in schizophrenia: Insights from affective neuroscience. Biological Psychiatry, 67, 899–901. 10.1016/j.biopsych.2010.02.022 20435208 PMC2864781

[bib95] Pizzagalli D. A., Holmes A. J., Dillon D. G., Goetz E. L., Birk J. L., Bogdan R., … Fava M. (2009). Reduced caudate and nucleus accumbens response to rewards in unmedicated individuals with major depressive disorder. American Journal of Psychiatry, 166, 702–710. 10.1176/appi.ajp.2008.08081201 19411368 PMC2735451

[bib96] Pizzagalli D. A., Jahn A. L., & O’Shea J. P. (2005). Toward an objective characterization of an anhedonic phenotype: A signal-detection approach. Biological Psychiatry, 57, 319–327. Retrieved from http://www.ncbi.nlm.nih.gov/entrez/query.fcgi?cmd=Retrieve&db=PubMed&dopt=Citation&list_uids=15705346 15705346 10.1016/j.biopsych.2004.11.026 PMC2447922

[bib97] Poldrack R. A. (2011). Inferring mental states from neuroimaging data: From reverse inference to large-scale decoding. Neuron, 72, 692–697. 10.1016/j.neuron.2011.11.001 22153367 PMC3240863

[bib98] Pulcu E., Trotter P. D., Thomas E. J., McFarquhar M., Juhasz G., Sahakian B. J., … Elliott R. (2014). Temporal discounting in major depressive disorder. Psychological Medicine, 44, 1825–1834. 10.1017/S0033291713002584 24176142 PMC4035754

[bib99] Pulcu E., Zahn R., Moll J., Trotter P. D., Thomas E. J., Juhasz G., … Elliott R. (2014). Enhanced subgenual cingulate response to altruistic decisions in remitted major depressive disorder. NeuroImage: Clinical, 4, 701–710. 10.1016/j.nicl.2014.04.010 24936421 PMC4053655

[bib100] Racagni G., Canonico P. L., Ravizza L., Pani L., & Amore M. (2004). Consensus on the use of substituted benzamides in psychiatric patients. Neuropsychobiology, 50, 134–143. 10.1159/000079104 15292667

[bib101] Radke S., Guths F., Andre J. A., Muller B. W., & de Bruijn E. R. (2014). In action or inaction? Social approach-avoidance tendencies in major depression. Psychiatry Research, 219, 513–517. 10.1016/j.psychres.2014.07.011 25060832

[bib102] Ratcliff R., Smith P. L., Brown S. D., & McKoon G. (2016). Diffusion decision model: Current issues and history. Trends in Cognitive Sciences, 20, 260–281. 26952739 10.1016/j.tics.2016.01.007 PMC4928591

[bib103] Raymond J. G., Steele J. D., & Series P. (2017). Modeling trait anxiety: From computational processes to personality. Frontiers in Psychiatry, 8, Article 1 10.3389/fpsyt.2017.00001 PMC5253387 28167920

[bib104] Rescorla R. A., & Wagner A. R. (1972). A theory of Pavlovian conditioning: Variations in the effectiveness of reinforcement and nonreinforcement. In Black A. H. & Prokasy W. F. (Eds.), Classical conditioning II (pp. 64–99). New York, NY: Appleton-Century-Crofts.

[bib105] Rizvi S. J., Pizzagalli D. A., Sproule B. A., & Kennedy S. H. (2016). Assessing anhedonia in depression: Potentials and pit falls. Neuroscience & Biobehavioral Reviews, 65, 21–35. 10.1016/j.neubiorev.2016.03.004 26959336 PMC4856554

[bib106] Robinson O. J., Bond R. L., & Roiser J. P. (2015). The impact of threat of shock on the framing effect and temporal discounting: Executive functions unperturbed by acute stress? Frontiers in Psychology, 6, Article 1315 10.3389/fpsyg.2015.01315 26441705 PMC4562307

[bib107] Robinson O. J., Cools R., Carlisi C. O., Sahakian B. J., & Drevets W. C. (2012). Ventral striatum response during reward and punishment reversal learning in unmedicated major depressive dis order. American Journal of Psychiatry, 169, 152–159. 10.1176/appi.ajp.2011.11010137 22420038 PMC5648982

[bib108] Robinson O. J., Frank M. J., Sahakian B. J., & Cools R. (2010). Dissociable responses to punishment in distinct striatal regions during reversal learning. Neuroimage, 51, 1459–1467. 10.1016/j.neuroimage.2010.03.036 20303408 PMC3038262

[bib109] Robinson O. J., Overstreet C., Charney D. S., Vytal K., & Grillon C. (2013). Stress increases aversive prediction error signal in the ventral striatum. Proceedings of the National Academy of Sciences of the United States of America, 110, 4129–4133. 10.1073/pnas.1213923110 23401511 PMC3593853

[bib110] Robinson O. J., Vytal K., Cornwell B. R., & Grillon C. (2013). The impact of anxiety upon cognition: Perspectives from human threat of shock studies. Frontiers in Human Neuroscience, 7, Article 203 10.3389/fnhum.2013.00203 PMC3656338 23730279

[bib111] Rock P. L., Roiser J. P., Riedel W. J., & Blackwell A. D. (2014). Cognitive impairment in depression: A systematic review and meta-analysis. Psychological Medicine, 44, 2029–2040. 10.1017/S0033291713002535 24168753

[bib112] Roiser J. P., Elliott R., & Sahakian B. J. (2012). Cognitive mechanisms of treatment in depression. Neuropsychopharmacology, 37, 117–136. 10.1038/npp.2011.183 21976044 PMC3238070

[bib113] Rothkirch M., Tonn J., Kohler S., & Sterzer P. (2017). Neural mechanisms of reinforcement learning in unmedicated patients with major depressive disorder. Brain, 140, 1147–1157. 10.1093/brain/awx025 28334960

[bib114] Schlagenhauf F., Rapp M. A., Huys Q. J., Beck A., Wustenberg T., Deserno L., … Heinz A. (2013). Ventral striatal prediction error signaling is associated with dopamine synthesis capacity and fluid intelligence. Human Brain Mapping, 34, 1490–1499. 10.1002/hbm.22000 22344813 PMC3731774

[bib115] Schultz W., Dayan P., & Montague P. R. (1997). A neural substrate of prediction and reward. Science, 275, 1593–1599. Retrieved from http://www.nc bi.nlm.nih.gov/pubmed/9054347 9054347 10.1126/science.275.5306.1593

[bib116] Segarra N., Metastasio A., Ziauddeen H., Spencer J., Reinders N. R., Dudas R. B., … Murray G. K. (2015). Abnormal frontostriatal activity during unexpected reward receipt in depression and schizophrenia: Relationship to anhedonia. Neuropsychopharmacology, 41, 2001–2010. 10.1038/npp.2015.370 26708106 PMC4820052

[bib117] Seymour B., Daw N., Dayan P., Singer T., & Dolan R. (2007). Differential encoding of losses and gains in the human striatum. Journal of Neuroscience, 27, 4826–4831. 10.1523/jneurosci.0400-07.2007 17475790 PMC2630024

[bib118] Shanks D. R., & St. John M. F. (1994). Characteristics of dissociable human learning-systems. Behavioral and Brain Sciences, 17, 367–395.

[bib119] Sherdell L., Waugh C. E., & Gotlib I. H. (2012). Anticipatory pleasure predicts motivation for reward in major depression. Journal of Abnormal Psychology, 121, 51–60. 10.1037/a0024945 21842963 PMC3335300

[bib120] Smoski M. J., Rittenberg A., & Dichter G. S. (2011). Major de pressive disorder is characterized by greater reward network activation to monetary than pleasant image rewards. Psychiatry Research, 194, 263–270. 10.1016/j.pscychresns.2011.06.012 22079658 PMC3225489

[bib121] Sokol-Hessner P., Hsu M., Curley N. G., Delgado M. R., Camerer C. F., & Phelps E. A. (2009). Thinking like a trader selectively reduces individuals’ loss aversion. Proceedings of the National Academy of Sciences of the United States of America, 106, 5035–5040. 19289824 10.1073/pnas.0806761106 PMC2656558

[bib122] Steele J. D., Kumar P., & Ebmeier K. P. (2007). Blunted response to feedback information in depressive illness. Brain, 130(Pt. 9), 2367–2374. 10.1093/brain/awm150 17586866

[bib123] Stoy M., Schlagenhauf F., Sterzer P., Bermpohl F., Hagele C., Suchotzki K., … Strohle A. (2012). Hyporeactivity of ven tral striatum towards incentive stimuli in unmedicated depressed patients normalizes after treatment with escitalopram. Journal of Psychopharmacology, 26, 677–688. 10.1177/0269881111416686 21926423

[bib124] Stringaris A., Vidal-Ribas Belil P., Artiges E., Lemaitre H., Gollier-Briant F., Wolke S., … Consortium I. (2015). The brain’s response to reward anticipation and depression in adolescence: Dimensionality, specificity, and longitudinal predictions in a community-based sample. American Journal of Psychiatry, 172, 1215–1223. 10.1176/appi.ajp.2015.14101298 26085042 PMC7614275

[bib125] Sutton R. S., & Barto A. G. (Eds.). (1998). Reinforcement learning: An introduction. Cambridge, MA: MIT Press.

[bib126] Taylor Tavares J. V., Clark L., Furey M. L., Williams G. B., Sahakian B. J., & Drevets W. C. (2008). Neural basis of abnormal response to negative feedback in unmedicated mood disorders. Neuroimage, 42, 1118–1126. 10.1016/j.neuroimage.2008.05.049 18586109 PMC2745889

[bib127] Treadway M. T., Bossaller N. A., Shelton R. C., & Zald D. H. (2012).Effort-based decision-making in major depressive dis order: A translational model of motivational anhedonia. Journal of Abnormal Psychology, 121, 553–558. 10.1037/a0028813 22775583 PMC3730492

[bib128] Treadway M. T., & Zald D. H. (2011). Reconsidering anhedonia in depression: Lessons from translational neuroscience. Neuroscience & Biobehavioral Reviews, 35, 537–555. 10.1016/j.neubiorev.2010.06.006 20603146 PMC3005986

[bib129] Tsetsos K., Gao J., McClelland J. L., & Usher M. (2012). Using time-varying evidence to test models of decision dynamics: Bounded diffusion vs. the leaky competing accumulator model. Frontiers in Neuroscience, 6, Article 79 10.3389/fnins.2012.00079 22701399 PMC3372959

[bib130] Tversky A., & Kahneman D. (1992). Advances in prospect theory: Cumulative representation of uncertainty. Journal of Risk and Uncertainty, 5, 297–323. 10.1007/BF00122574

[bib131] Ubl B., Kuehner C., Kirsch P., Ruttorf M., Diener C., & Flor H. (2015). Altered neural reward and loss processing and prediction error signalling in depression. Social Cognitive & Affective Neuroscience, 10, 1102–1112. 10.1093/scan/nsu158 25567763 PMC4526479

[bib132] Vadillo M. A., Konstantinidis E., & Shanks D. R. (2016). Under powered samples, false negatives, and unconscious learning. Psychonomics Bulletin & Reviews, 23, 87–102. 10.3758/s13423-015-0892-6 PMC4742512 26122896

[bib133] Vallesi A., Canalaz F., Balestrieri M., & Brambilla P. (2015). Modulating speed-accuracy strategies in major depression. Journal of Psychiatric Research, 60, 103–108. 10.1016/j.jpsychires.2014.09.017 25294698

[bib134] van Ravenzwaaij D., Donkin C., & Vandekerckhove J. (2017). The EZ diffusion model provides a powerful test of simple empirical effects. Psychonomics Bulletin & Reviews, 24, 547–556. 10.3758/s13423-016-1081-y PMC5389995 27352898

[bib135] Vrieze E., Pizzagalli D. A., Demyttenaere K., Hompes T., Sienaert P., de Boer P., … Claes S. (2012). Reduced reward learning predicts outcome in major depressive disorder. Biological Psychiatry, 73, 639–645. 10.1016/j.biopsych.2012.10.014 23228328 PMC3602158

[bib136] Wacker J., Dillon D. G., & Pizzagalli D. A. (2009). The role of the nucleus accumbens and rostral anterior cingulate cortex in anhedonia: Integration of resting EEG, fMRI, and volumetric techniques. Neuroimage, 46, 327–337. 10.1016/j.neuroimage.2009.01.058 19457367 PMC2686061

[bib137] White C. N., Ratcliff R., Vasey M. W., & McKoon G. (2010). Using diffusion models to understand clinical disorders. Journal of Mathematical Psychology, 54, 39–52. 20431690 10.1016/j.jmp.2010.01.004 PMC2859713

[bib138] Whitmer A. J., Frank M. J., & Gotlib I. H. (2012). Sensitivity to reward and punishment in major depressive disorder: Effects of rumination and of single versus multiple experiences. Cognition & Emotion, 26, 1475–1485. 10.1080/02699931.2012.682973 22716241 PMC11880990

[bib139] Wilson R. C., Geana A., White J. M., Ludvig E. A., & Cohen J. D. (2014). Humans use directed and random exploration to solve the explore-exploit dilemma. Journal of Experimental Psychology, General, 143, 2074–2081. 10.1037/a0038199 25347535 PMC5635655

[bib140] Wu C. C., Samanez-Larkin G. R., Katovich K., & Knutson B. (2014). Affective traits link to reliable neural markers of incentive anticipation. Neuroimage, 84, 279–289. 10.1016/j.neuroimage.2013.08.055 24001457 PMC3849140

[bib141] Xu J. (2015). Implications of cortical balanced excitation and inhibition, functional heterogeneity, and sparseness of neuronal activity in fMRI. Neuroscience & Biobehavioral Reviews, 57, 264–270. 10.1016/j.neubiorev.2015.08.018 26341939 PMC4623927

[bib142] Yang X. H., Huang J., Lan Y., Zhu C. Y., Liu X. Q., Wang Y. F., … Chan R. C. (2016). Diminished caudate and superior temporal gyrus responses to effort-based decision making in patients with first-episode major depressive disorder. Progress in Neuropsychopharmacology & Biological Psychiatry, 64, 52–59. 10.1016/j.pnpbp.2015.07.006 26192817

[bib143] Yang X. H., Huang J., Zhu C. Y., Wang Y. F., Cheung E. F., Chan R. C., & Xie G. R. (2014). Motivational deficits in effort-based decision making in individuals with subsyndromal depression, first-episode and remitted depression patients. Psychiatry Research, 220, 874–882. 10.1016/j.psychres.2014.08.056 25262638

[bib144] Yonkers K. A., Warshaw M. G., Massion A. O., & Keller M. B. (1996). Phenomenology and course of generalised anxiety disorder. British Journal of Psychiatry, 168, 308–313. 10.1192/bjp.168.3.308 8833684

[bib145] Yuille A. L., & D. Gaiger (2003). Winner-take-all networks. In Arbib M. A., (Ed.), The handbook of brain theory and neural networks (2nd ed., pp. 1228–1231). Cambridge, MA: MIT Press.

[bib146] Zhang W. N., Chang S. H., Guo L. Y., Zhang K. L., & Wang J. (2013). The neural correlates of reward-related processing in major depressive disorder: A meta-analysis of functional magnetic resonance imaging studies. Journal of Affective Disorders, 151, 531–539. 10.1016/j.jad.2013.06.039 23856280

